# Hemodynamic Induction of Intracranial Aneurysms via Bilateral Common Carotid Artery Ligation in Rabbits: A Systematic Review and Meta-Analysis

**DOI:** 10.3390/medsci14060645

**Published:** 2026-10-07

**Authors:** Laurentiu Andrei Blaj, Victor Ianole, Andrei Ionut Cucu, Bogdan-Ionel Tamba, Gabriela-Dumitrita Stanciu, Mihaela Dana Turliuc

**Affiliations:** 1 Department of Neurosurgery, Grigore T. Popa University of Medicine and Pharmacy, 700115 Iasi, Romania; laurentiu.blaj@umfiasi.ro (L.A.B.); mihaela.turliuc@umfiasi.ro (M.D.T.); 2 Department of Pathology, Grigore T. Popa University of Medicine and Pharmacy, 700115 Iasi, Romania; 3 Department of Biomedical Sciences, Faculty of Medicine and Biological Sciences, “Stefan cel Mare” University, 720229 Suceava, Romania; andrei.cucu@usm.ro; 4 Advanced Centre for Research-Development in Experimental Medicine “Prof. Ostin C. Mungiu”—CEMEX, Grigore T. Popa University of Medicine and Pharmacy, 700115 Iasi, Romania; bogdan.tamba@umfiasi.ro (B.-I.T.); gabriela-dumitrita.stanciu@umfiasi.ro (G.-D.S.)

**Keywords:** intracranial aneurysm, bilateral common carotid artery ligation, rabbits, internal elastic lamina, vascular remodeling, hemodynamics

## Abstract

Background/Objectives: The rabbit bilateral common carotid artery ligation (BCCAL) model is utilized to simulate hemodynamically driven intracranial aneurysm formation. However, inconsistent reporting and heterogeneous endpoints obscure its baseline efficacy. This systematic review and meta-analysis determines the pooled incidence of aneurysm induction and maps the temporal progression of associated structural and hemodynamic changes. Methods: A search of PubMed, Embase, and Web of Science (through 5 September 2026) identified experimental studies implementing the BCCAL model. We evaluated macroscopic aneurysm formation using a random-effects generalized linear mixed model (GLMM). Destructive aneurysmal remodeling, for which GLMM was not statistically appropriate due to a uniform event rate, was assessed as an exact binomial proportion. Secondary outcomes (mortality, hemodynamics, and histopathological degradation) were assessed utilizing single-arm continuous, comparative binary, and multilevel mixed-effects meta-analyses, with cluster-robust sensitivity analyses where repeated measures were present. Results: Across 22 included studies, the pooled incidence of macroscopic aneurysm formation was 23.4% (k = 3 studies), while destructive structural injury was reported in all animals examined for that outcome, though its operational definition varied across studies. BCCAL increased absolute mortality risk by 16.6% compared with sham controls. Acutely (1 to 7 days), basilar blood flow velocity and WSS rose significantly, accompanying rapid internal elastic lamina (IEL) destruction (mean gap 507.34 µm) and media thinning (450.32 µm). Flow rate also increased, though this trend did not survive cluster-robust sensitivity analysis. In the chronic phase (>7 days), compensatory vasodilatation attenuated localized hemodynamic stress. Whether the structural defect subsequently remained stable or progressed further could not be conclusively determined. Conclusions: The rabbit BCCAL model reliably induces early-stage aneurysmal remodeling via a rapid mechanical insult, though its long-term structural trajectory remains unresolved. This study provides preliminary incidence and mortality estimates to help inform sample size and power calculations for future preclinical neurovascular trials.

## 1. Introduction

Unruptured intracranial aneurysms (IAs) are common in the general population, with an estimated prevalence of 0.4–6% and a rupture rate of approximately 0.7% if left untreated [1]. The pathophysiology regarding the formation and progression of IAs is not fully understood, which is why researchers have relied on preclinical animal models to replicate them and to test new pharmacological treatments [2]. One hypothesis is that high hemodynamic environments, such as systemic hypertension or atherosclerosis, lead to vessel wall inflammation with subsequent remodeling that may trigger aneurysm formation [3].

The BCCAL rabbit model was designed to exploit this hemodynamic hypothesis. First designed by Hassler in 1963, the surgical occlusion of the anterior circulation forces a compensatory surge of blood flow into the posterior circulation [4]. Rabbits are particularly suited for this intervention because the anatomical geometry of their posterior circulation allows for the hemodynamic shift to focus high WSS at the basilar terminus. This contrasts with rodent models, where rats have a larger Circle of Willis relative to the basilar artery (BA), more branching vessels, and steeper bifurcation angles [5]. Because rabbits lack this protective geometry, the BCCAL model better simulates the pathophysiological mechanisms underlying aneurysm initiation in humans, thereby driving localized arterial degradation, IEL fragmentation, and medial thinning.

Despite its application over the last six decades, reported success rates for the BCCAL model are highly heterogeneous across the literature. Published induction rates range from 100% in some laboratories to only a few cases of macroscopic aneurysm formation in others. The discrepancy is largely driven by the lack of standardized reporting and varying definitions of what constitutes a “successful” aneurysm. While some authors are satisfied with medial bulging, others report mature saccular lesions. This difference is making it difficult for investigators to perform power analyses to determine the appropriate sample sizes required for experimental designs. Furthermore, it is unclear whether the aneurysm induction occurs immediately after ligation or if the rabbit artery slowly degenerates over time.

To our knowledge, this is the first systematic review and meta-analysis to address these methodological and pathophysiological issues. Our primary goal is to estimate the pooled incidence of both macroscopic aneurysm formation and destructive aneurysmal remodeling associated with the BCCAL model. Secondarily, we aim to map the temporal progression of the induced morphological and hemodynamic changes, while evaluating the methodological quality, reporting transparency, and risk of bias across literature to establish a baseline for future experimental designs.

## 2. Materials and Methods

### 2.1. Study Design

This systematic review and meta-analysis were conducted in accordance with PRISMA guidelines. Details of the protocol have been uploaded for registration on the International Prospective Register of Systematic Reviews (PROSPERO 2026 CRD420261424551).

### 2.2. Search Strategy

We selected relevant studies from publications in PubMed, Embase and Web of Science databases from inception until 1 May 2026, and subsequently again until 5 September 2026, to ensure up-to-date evidence. No additional filter was applied for study type and publication year. The search strategy was developed by combining three primary concept blocks using standard Boolean operators. The specific domains searched included animal model (e.g., rabbits), experimental intervention (e.g., bilateral common carotid artery ligation), and the target anatomical and pathological outcomes (e.g., intracranial aneurysm, basilar terminus, vascular remodeling, WSS, and IEL degradation). The full, reproducible Boolean search syntax for all databases is provided in the Supplementary Material.

### 2.3. Study Selection: Inclusion and Exclusion Criteria

The inclusion criteria are represented by studies on rabbits of any strain, age or sex that were used for the implementation of a hemodynamic model of bilateral common carotid artery ligation to induce cerebrovascular stress. The studies had to have at least one sham-operated or naive rabbit group and at least one group with the intervention.

Studies were included if they implemented the BCCAL model in rabbits and reported at least one of our pre-specified primary or secondary outcomes. Studies reporting only secondary hemodynamic, morphometric, or histopathological endpoints, without data on aneurysm incidence itself, were therefore eligible. This represents a deviation from the protocol registered at PROSPERO (CRD420261424551), which specified that eligible studies report the primary composite outcome. In practice, and as reflected in Table S1, eligibility was extended to studies reporting only the pre-specified secondary endpoints, in order to capture the full body of available evidence on the model’s biological effects. This deviation did not affect the composition of studies contributing to either co-primary outcome.

To accurately capture the distinct stages of the pathophysiological continuum for studies reporting aneurysm incidence, we defined two co-primary outcomes: Primary Outcome A (Macroscopic Aneurysm Formation) is defined as the incidence of structurally mature, definitive saccular or fusiform IAs located at the Circle of Willis, and Primary Outcome B (Destructive Aneurysmal Remodeling) is defined as the incidence of localized structural degradation, such as outward medial bulges or isolated destructive vessel wall changes that have not progressed to mature macroscopic aneurysms.

Secondary outcomes included isolated qualitative or quantitative markers of aneurysmal remodeling. These included hemodynamic parameters (Peak Flow Velocity, WSS, WSS Gradient), vascular morphometrics and volumetric data (BA Volume, Basilar Vasodilatation Index (VDI), luminal diameter), histopathological markers of structural integrity (fragmentation/loss of the IEL, Aneurysm Development Score (ADS), tunica media thickness), and mortality events.

Regarding the exclusion criteria, only original experimental articles published in peer-reviewed journals were included. Review articles, editorials and conference abstracts were excluded. The search was limited to articles published in the English language. Also, studies that lack the necessary data for quantitative meta-analysis and cannot be contacted to provide the raw data, but that comply with the inclusion criteria, were used only for narrative synthesis.

### 2.4. Data Extraction

The following details were extracted by two reviewers (L.A.B. and A.I.C.) independently from each study: (1) document elements: title, authors, year of publication; (2) experimental animal elements: animal species, sex, age, weight; (3) intervention elements: experimental sample size, anesthesia, follow-up; (4) experimental outcomes: incidence of macroscopic aneurysm formation, incidence of destructive aneurysmal remodeling, mortality, WSS, WSSG, blood flow velocity, BA flow rate, BA diameter, VDI, BA vessel volume, IEL loss, media thinning and ADS.

After exporting each database record, the retrieved articles were imported into EndNote v20. After eliminating duplicate articles, two independent reviewers (L.A.B. and A.I.C.) examined the articles in a two-step process: screening titles and abstracts, followed by a full-text assessment to ensure they met the required criteria. Any conflicts between the two reviewers were resolved by a third researcher (M.D.T.).

### 2.5. Quality Assessment

Quality assessment of the studies was conducted by two independent reviewers (L.A.B. and V.I.). Any discrepancies were resolved through consultation with a third reviewer (B.-I.T.). The methodological quality and reporting transparency were evaluated using the Systematic Review Centre for Laboratory Animal Experimentation (SYRCLE) Risk of Bias tool for animal studies and the Animals Research: Reporting in Vivo Experiments (ARRIVE) guidelines for reporting animal experiments.

### 2.6. Statistical Analysis

Because this meta-analysis evaluates two distinct co-primary outcomes (Macroscopic Aneurysm Formation and Destructive Aneurysmal Remodeling), a statistical adjustment for multiplicity was required to control the family-wise error rate and prevent Type I errors. In accordance with standard methodological guidelines for multiple primary endpoints, we adjusted our threshold for statistical significance to α = 0.025. Consequently, all pooled proportional estimates and respective variances for the co-primary outcomes are reported using 97.5% Confidence Intervals (CIs). A *p*-value of less than 0.025 was considered statistically significant for these primary analyses. For all secondary outcomes and continuous parameters, the standard α = 0.05 threshold and 95% CIs were maintained.

A single-arm continuous meta-analysis was utilized to pool the raw means across the experimental BCCAL cohorts since all healthy control subjects exhibited zero statistical variance. A comparative binary meta-analysis utilizing the Risk Difference (RD) was conducted to estimate the absolute risk increase. Because RD remains mathematically computable even when a cell frequency is zero, unlike ratio measures such as risk ratio or odds ratio, no continuity correction was applied to the primary mortality analysis. A sensitivity analysis incorporating the conventional 0.5 continuity correction was additionally performed to confirm the robustness of this choice.

For continuous macroscopic outcomes evaluated at isolated, independent timepoints without repeated measures, a standard continuous pairwise meta-analysis was performed. The pooled effect size was calculated as the Mean Difference (MD) using the restricted maximum-likelihood (REML) estimator for between-study variance.

For Primary Outcome A (Macroscopic Aneurysm Formation), the pooled incidence was estimated using a random-effects logistic GLMM, with the Maximum Likelihood (ML) estimator used to calculate the between-study variance component. For Primary Outcome B (Destructive Aneurysmal Remodeling), where this approach was not appropriate (every contributing study had a 100% event rate, producing complete separation in the logistic model), the pooled incidence was instead calculated directly as an exact (Clopper-Pearson) binomial proportion on the aggregate event count, which does not require estimating a variance component.

To analyze continuous hemodynamic parameters extracted from longitudinal studies, a standard pooling approach was avoided to prevent the statistical double-counting of animals subjected to repeated measures. Instead, a Multilevel Mixed-Effects Meta-Analysis was executed. Temporal observations were nested within their true experimental base cohorts as random-effects components, properly partitioning within-cohort and between-cohort variance while pooling the MD. Between-study heterogeneity was assessed using Cochran’s Q statistics and, where possible, quantified using Higgins’ I-square index. For pairwise comparisons based on only two contributing studies, I^2^ is not a reliably estimable quantity and must be therefore interpreted with care.

Because several base studies contributed multiple longitudinal timepoints compared against the same underlying control cohort, the sampling errors of these repeated effect sizes are likely correlated beyond what a random intercept alone captures. This correlation cannot be estimated directly from the summary-level data reported in the source publications, which provide no measure of within-animal repeatability across visits. As a transparent sensitivity check, cluster-robust variance estimation (CR2 small-sample correction with Satterthwaite degrees of freedom, clustered at the base-study level) was additionally applied to each multilevel hemodynamic model, to verify that the direction and significance of the pooled estimates were not an artifact of unmodeled within-cluster correlation. To evaluate the temporal dynamics of the aneurysm model, dual longitudinal analyses were performed. First, categorical subgroup analyses were conducted by stratifying the data into acute (1 to 7 days post-ligation) and subacute/chronic (greater than 7 days) clinical phases. Subgroup differences were evaluated using the Omnibus test of moderators. Second, continuous mixed-effects meta-regression was applied using post-ligation follow-up days as a continuous predictive moderator to calculate exact daily progression and decay. All statistical analyses were performed using R statistical software.

Outcomes reported by an insufficient number of studies to permit mathematical pooling, or those utilizing heterogeneous, incompatible descriptive grading scales, were evaluated through a structured narrative synthesis.

## 3. Results

### 3.1. Study Selection Process

**Figure 1:**
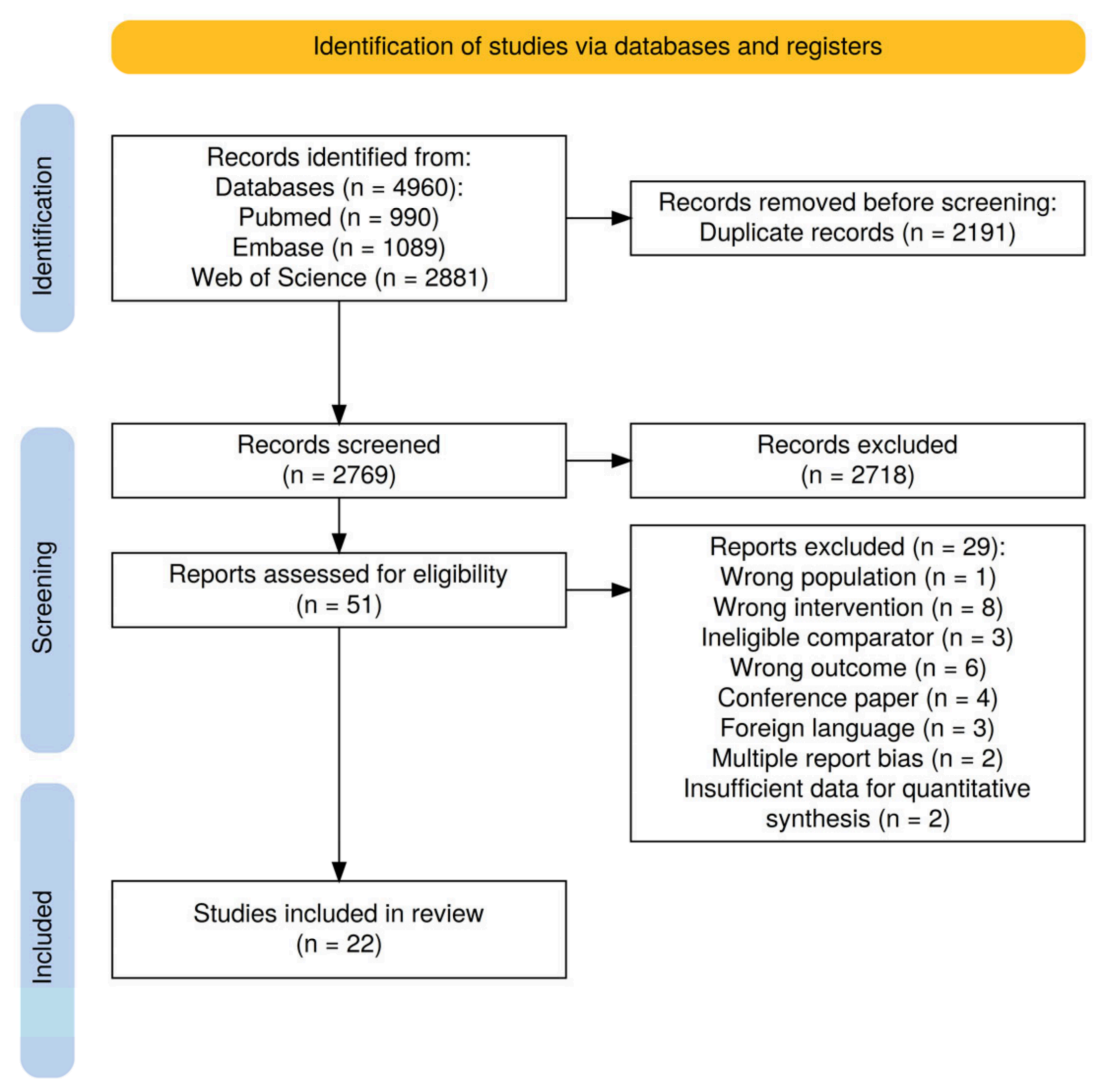
PRISMA flow diagram.

We initially identified 4960 records from the three databases. After removing 2191 duplicate records, 2769 studies were screened by two reviewers (L.A.B. and A.I.C.) based on title and abstract searching for predefined exclusion criteria. This resulted in 2718 records being excluded. The remaining 51 articles were assessed in full text for eligibility, and 29 reports were excluded. In the end, the meta-analysis was conducted using data from 22 studies. The PRISMA flowchart of the study selection process is shown in Figure 1.

### 3.2. Study Characteristics

A summary of the study characteristics of the included studies is displayed in Table 1. Studies were published between 1963 and 2021. Most studies used New Zealand White rabbits (*n* = 17); one study used the Japanese White strain, one used hybrid rabbits, and 3 did not report the strain. Female rabbits were predominantly used (12 vs. 7 studies); however, the aggregate animal count shows a male bias (293 male vs. 183 female), because Wu 2015 [6] included half of the cohort (144 animals). An additional 91 animals were used in 3 studies that failed to report sex entirely. Regarding age, 10 studies reported their subjects simply as “Adult” rather than providing a specific age. Seven studies did not report the age of the animals. The remaining studies reported ages typically ranging from 4 to 8 months, except for Hassler 1963 [4], who utilized a mixed-age cohort spanning from 3 weeks to 1 year. The weight of the animals was reported to be within 2 to 5 kg, however 4 studies failed to report the animals’ weight entirely.

Across the included literature, a total of 567 rabbits were utilized to investigate the experimental outcomes as follows: 260 animals were assigned to the primary BCCAL intervention groups, while baseline comparisons, such as sham and controls, used 136 and 39 animals, respectively. The remaining rabbits were assigned to alternative pharmacological or surgical intervention groups that fall outside the scope of this meta-analysis. The induction and maintenance of anesthesia were predominantly achieved using a combination of ketamine and xylazine, supplemented with isoflurane across 7 studies. 6 studies failed to report the anesthetic agents utilized during their experimental procedures.

Regarding the follow-up period, 5 studies looked at the immediate effects of BCCAL in the first 1 to 5 days, while all the rest (*n* = 17) had various periods ranging from 1 to 6 months with a mean follow-up of 90.88 days.

Table S1 (Supplementary Materials) summarizes which of the 22 included studies contributed data to each quantitative outcome. Two studies (Tutino 2013 [5], Fumoto 2021 [7]) were included only in the narrative synthesis of BA diameter and tortuosity index findings and did not contribute to any pooled quantitative analysis.

**Table 1:** General Characteristics of the included studies. NZW = New Zealand White, F = Female, M = Male, NR = Not Reported.

Study, Year	Strain	Sex	Age (Reported)	Weight (Reported)	Total	Control	Sham	BCCAL	Follow-Up (Reported)	Anesthesia/Pre-meds
Aydin 2020 [8]	NR	NR	NR	NR	24	5	5	14	2 months (60 days)	Isoflurane; Ketamine and Xylazine
Aygul 2013 [9]	NR	NR	NR	NR	25	5	5	15	2 months (60 days)	NR
Eseoglu 2014 [10]	NZW	M	NR	3.7 ± 0.4 kg	21	NR	5	8	2 months (60 days)	Isoflurane; Ketamine HCl; Xylazine HCl; Metamizole
Fumoto 2021 [7]	Japanese White	F	6 months	3.8–4.5 kg	10	NR	4	6	12 weeks (84 days)	Medetomidine 0.5 mg/kg; Midazolam 2 mg/kg; Butorphanol 0.5 mg/kg
Gao 2008 [11]	NZW	F	Adult	3–4 kg	9	NR	3	3	13 weeks (84 days)	Ketamine and xylazine
Hassler 1963 [4]	NR	NR	Mixed (3 weeks vs. 1 year)	NR	42	10	NR	2	1 to 5 months (150 days)	NR
Hoi 2008 [12]	NZW	F	Adult	3–4 kg	11	NR	3	5	0 to 84 Days	Ketamine and xylazine
Kilic 2019 [13]	NZW	M	Adult	3.7 ± 0.4 kg	23	5	5	13	1 month (30 days)	Isoflurane; Ketamine and Xylazine (s.c.)
Kolega 2011 [14]	NZW	F	Adult	3–4 kg	18	NR	5	13	2 to 5 days	NR
Kolega 2021 [15]	NZW	F	NR	4–5 kg	4	NR	2	2	1 day	NR
Li 2014 [16]	NZW	F	Adult	3–4 kg	15	NR	5	10	0 to 84 Days	Ketamine and xylazine
Liaw 2014 [17]	NZW	F	6–8 months	NR	19	NR	5	7	5 days	Ketamine and xylazine
Mandelbaum 2013 [18]	NZW	F	6–8 months	4–5 kg	45	NR	7	17	5 days to 6 months (180 days)	Ketamine; Xylazine; Isoflurane (2%)
Meng 2011 [19]	NZW	F	Adult	3–4 kg	25	NR	5	20	27 weeks (189 days)	NR
Metaxa 2010 [20]	NZW	F	Adult	3–4 kg	11	NR	3	8	5 days	Ketamine i.m. 35 mg/kg; Xylazine i.m. 5 mg/kg; Isoflurane 2%
Özkan 2004 [21]	Hybrid	M	NR	2.5–3 kg	20	5	NR	15	4 months (120 days)	Ketamine hydrochloride IM 50 mg/kg
Tremmel 2010 [22]	NZW	F	Adult	3–4 kg	4	NR	1	3	5 days	NR
Tutino 2013 [5]	NZW	F	NR	4–5 kg	12	NR	3	9	5 days to 6 months (180 days)	Ketamine and Xylazine (from Gao et al.)
Wu 2015 [6]	NZW	M	4 months	2.0–2.5 kg	144	NR	48	48	1 to 4 weeks (30 days)	Xylazine 0.2 mL/kg i.m.; Pentobarbital 20 mg/kg
Wu 2017 [23]	NZW	M	NR	2.5–3.5 kg	36	NR	12	12	1 to 4 weeks (30 days)	Xylazine hydrochloride 0.2 mL/kg intraperitoneal; Sodium Pentobarbital 1%, 20 mg/kg intraperitoneal
Yilmaz 2016 [24]	NZW	M	Adult	3.7 ± 0.4 kg	24	4	5	15	2 months (60 days)	Isoflurane 0.2 mL/kg; Ketamine HCl (150 mg/1.5 mL) and Xylazine HCl (30 mg/1.5 mL) subcutan
Yilmaz 2017 [25]	NZW	M	Adult	3.7 ± 0.4 kg	25	5	5	15	2 months (60 days)	Isoflurane 0.2 mL/kg; Ketamine HCl (150 mg/1.5 mL) and Xylazine HCl (30 mg/1.5 mL) subcutan

### 3.3. Risk of Bias and Reporting Quality Assessment

An analysis of the ARRIVE guidelines revealed a distinct reporting profile characteristic of the rabbit BCCAL model literature. The included studies demonstrated compliance in areas concerning basic experimental setup and ethics. Nearly all studies (>95%) explicitly reported obtaining institutional ethical approval and provided highly detailed, reproducible methodologies regarding surgical techniques, anesthesia protocols, and ligation materials. Furthermore, basic animal demographics, specifically strain (predominantly New Zealand White) and sex, were consistently reported across the literature (86.4%). However, critical gaps in reporting transparency were identified in the domains of experimental rigor and bias mitigation. Most notably, no study reported conducting a sample size calculation or power analysis to mathematically justify the number of animals utilized. Furthermore, while most studies claimed that animals were “randomly allocated,” only 13.6% reported the specific method used to generate the sequence. Finally, explicit inclusion and exclusion criteria were rarely stated (18.2%), with animal exclusions frequently occurring as post hoc reactions to procedural mortality rather than following predefined protocols.

**Figure 2:**
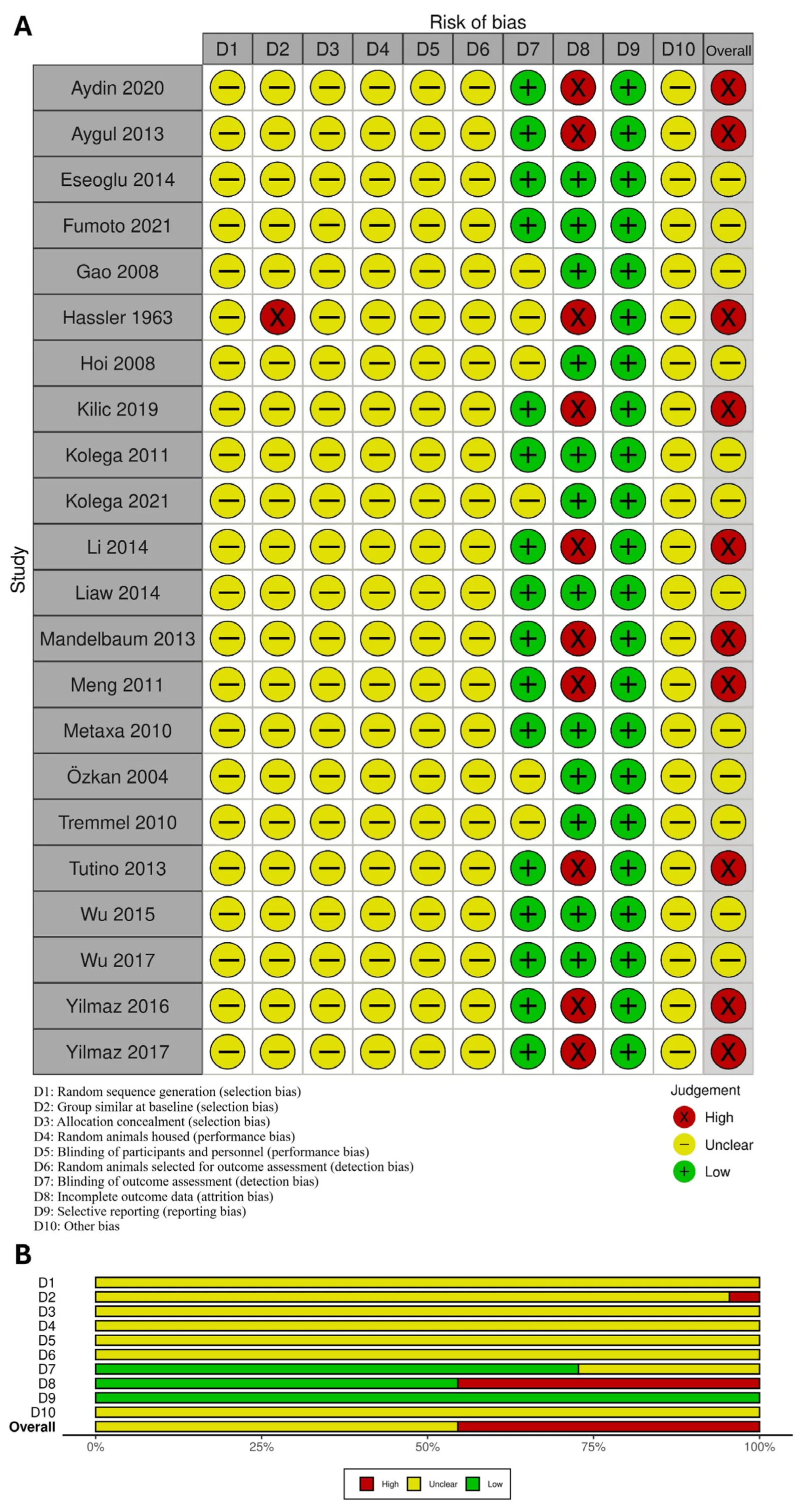
Risk-of-bias assessment for the included experimental cohorts, evaluated using the SYRCLE tool. (**A**) Traffic light plot detailing the risk-of-bias judgments for each individual study across the 10 assessed domains (D1–D10; domains are listed below the plot): green circle with “+” = low risk of bias; yellow circle with “−” = unclear risk of bias; red circle with “X” = high risk of bias; “Overall” = overall judgment per study. (**B**) Summary bar plot illustrating the proportion of studies categorized as low (green), unclear (yellow) or high (red) risk of bias for each domain and overall. Study labels give the first author and year: Aydin 2020 [8]; Aygul 2013 [9]; Eseoglu 2014 [10]; Fumoto 2021 [7]; Gao 2008 [11]; Hassler 1963 [4]; Hoi 2008 [12]; Kilic 2019 [13]; Kolega 2011 [14]; Kolega 2021 [15]; Li 2014 [16]; Liaw 2014 [17]; Mandelbaum 2013 [18]; Meng 2011 [19]; Metaxa 2010 [20]; Özkan 2004 [21]; Tremmel 2010 [22]; Tutino 2013 [5]; Wu 2015 [6]; Wu 2017 [23]; Yilmaz 2016 [24]; Yilmaz 2017 [25].

These reporting failures translated directly into an “Unclear” risk of bias across most SYRCLE domains, particularly regarding selection and performance biases (Figure 2). Although detection bias was effectively mitigated through the consistent use of blinded stereology experts, significant methodological concerns emerged regarding attrition bias, as acute procedural mortalities were frequently excluded from final analyses rather than appropriately accounted for. Consequently, despite highly standardized surgical execution, the overall quality of the evidence must be downgraded to moderate-to-low due to these systemic methodological and reporting shortcomings. Formal statistical testing for publication bias (e.g., funnel plot asymmetry or Egger’s regression test) was not performed, as the number of studies contributing to any individual quantitative outcome (ranging from k = 2 to k = 7) was insufficient to yield a reliable or interpretable assessment. Methodological quality and reporting transparency were instead evaluated through the SYRCLE and ARRIVE frameworks described above.

### 3.4. Primary Outcome Assessment: Aneurysm Incidence

#### 3.4.1. Primary Outcome A (Macroscopic Aneurysm Formation)

**Figure 3:**
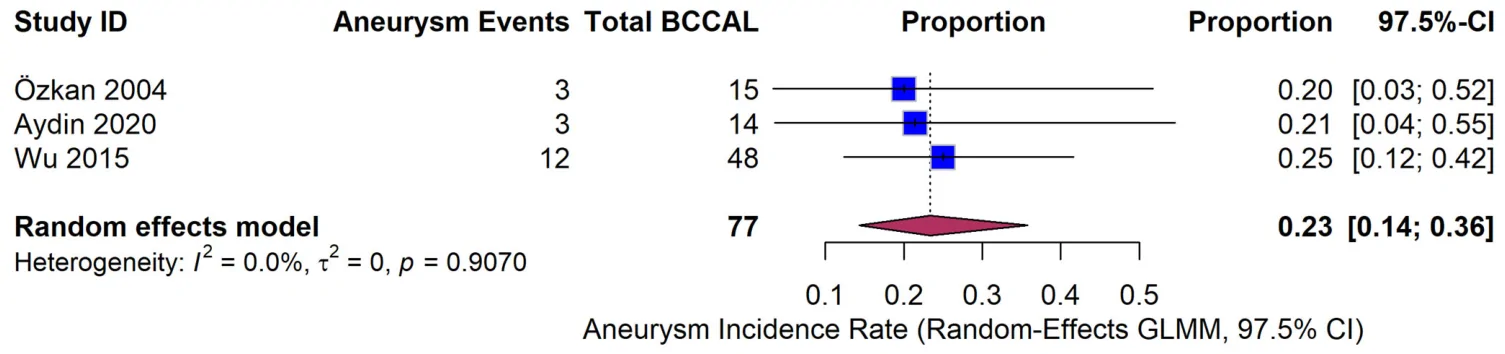
Forest plot displaying the pooled incidence of macroscopic aneurysm formation in the BCCAL rabbit model. Events = number of animals with macroscopic aneurysm formation; Total BCCAL = number of BCCAL animals per study. Blue squares: incidence proportion of each study (square size proportional to its weight); horizontal lines: 97.5% confidence intervals (adjusted for the two co-primary outcomes); maroon diamond: pooled incidence from the random-effects logistic GLMM (diamond width = 97.5% CI). Study labels give the first author and year: Özkan 2004 [21]; Aydin 2020 [8]; Wu 2015 [6].

A total of 3 studies, encompassing 77 BCCAL rabbit models, reported on the incidence of macroscopic aneurysm formation. Across these cohorts, 18 aneurysm events were observed. The pooled incidence was estimated using a random-effects logistic generalized linear mixed model. The pooled incidence of aneurysm formation in BCCAL rabbits was 23.4% (97.5% CI: 14.3% to 35.8%; Figure 3).

#### 3.4.2. Primary Outcome B (Destructive Aneurysmal Remodeling)

All seven studies reporting on destructive aneurysmal remodeling documented evidence of localized structural injury, though the operational definition varied across laboratories: three studies (Gao 2008 [11], Hassler 1963 [4], Liaw 2014 [17]) confirmed a macroscopic medial bulge, while the remaining four (Kolega 2011 [14], Li 2014 [16], Meng 2011 [19], Metaxa 2010 [20]) based the diagnosis on histological or molecular markers of wall injury without necessarily describing a gross lesion (Table 2). Given the complete absence of between-study variation in event status (60 of 60 events across 7 studies), a pooled exact binomial proportion was calculated directly on the aggregate event count. The exact (Clopper-Pearson) pooled incidence was 100% (97.5% CI: 93% to 100%). Precision varied considerably with study size: the lower bound of the 97.5% CI was 11% for the smallest cohort (Hassler 1963 [4], 2/2) and 77% for the largest (Meng 2011 [19], 17/17). This underscores that the flat 100% aggregate conceals substantial uncertainty at the individual-study level. This finding is best read as confirmation that the BCCAL model reliably induces detectable, aneurysm-like structural injury, rather than as evidence of a uniform lesion phenotype or severity across laboratories.

### 3.5. Secondary Outcome Assessment

#### 3.5.1. Mortality in BCCAL Rabbit Models

**Figure 4:**
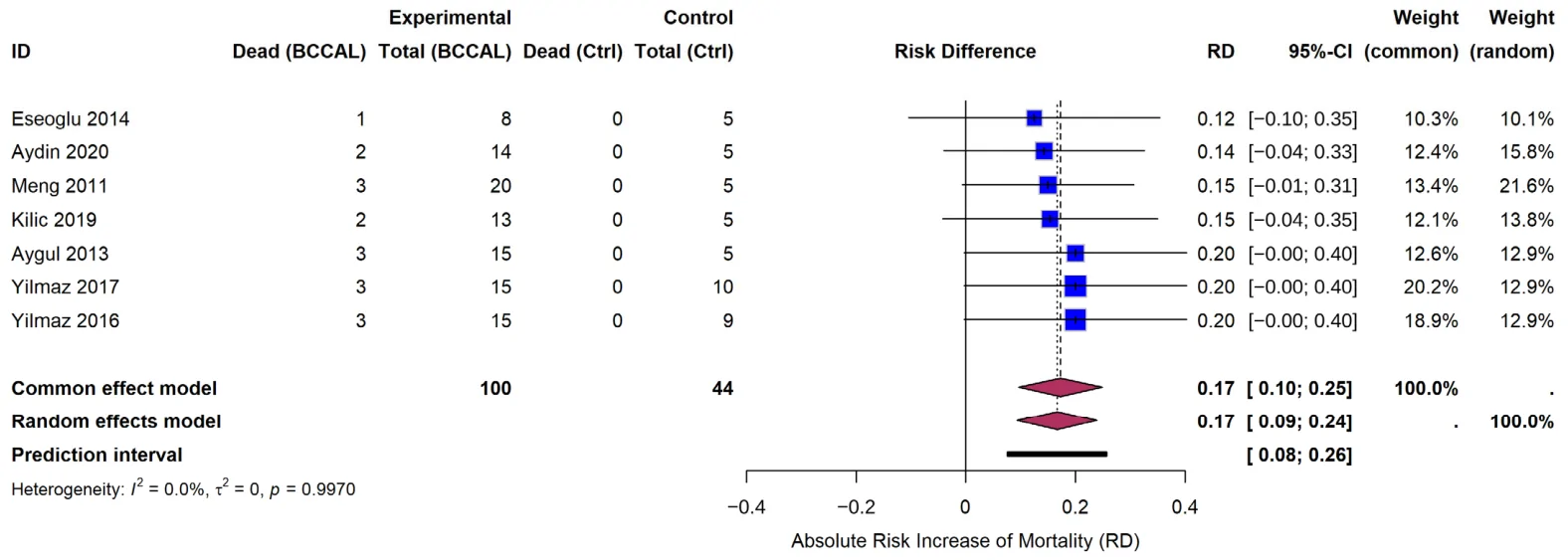
Meta-analysis of the mortality rate. Forest plot of the risk difference (RD) in mortality between BCCAL and control animals (no continuity correction). Dead = number of animals that died; Total = number of animals per group (Experimental = BCCAL; Ctrl = control group, including sham-operated animals). Blue squares: RD of each study (square size proportional to its weight); horizontal lines: 95% confidence intervals; maroon diamonds: pooled common-effect and random-effects estimates (diamond width = 95% CI); thick black bar: 95% prediction interval; solid vertical line at 0: no difference in mortality between groups; dotted vertical lines: pooled estimates (RD = 0.17). Weights (%) are shown for both models (“.” = not applicable). I^2^ and τ^2^ = heterogeneity statistics. Study labels give the first author and year: Eseoglu 2014 [10]; Aydin 2020 [8]; Meng 2011 [19]; Kilic 2019 [13]; Aygul 2013 [9]; Yilmaz 2016 [24]; Yilmaz 2017 [25].

To define the safety threshold and procedural attrition of the BCCAL model, a comparative meta-analysis evaluating mortality was conducted across 7 studies comprising 144 rabbits. Among the 100 rabbits subjected to the intervention, the mortality rate was 17%, as shown in Figure 4. Cochran’s Q test and the I^2^ index indicated low statistical heterogeneity among the reporting laboratories (Q = 0.56, df = 6, *p* = 0.9970; I^2^ = 0%, 95% CI: 0.0% to 70.8%). Given the width of this confidence interval, this should be read as evidence of low observed heterogeneity in this sample rather than proof that mortality risk is identical across all experimental designs. The control arms, consisting of sham-operated animals, reported 0% mortality out of 44 animals. Between-study variance was non-existent, and the common-effect and random-effects models converged to an identical estimate.

The pooled random-effects RD was calculated at +0.1663 (95% CI: 0.0935 to 0.2390). This indicates a statistically significant 16.6% increase in mortality risk for rabbits undergoing BCCAL compared with sham controls (z = 4.48, *p* < 0.0001). No continuity correction was applied to this primary estimate, since Risk Difference remains computable without one. A sensitivity analysis applying the conventional 0.5 correction yielded a closely consistent point estimate (RD = 0.1720, 95% CI: 0.0675 to 0.2766, z = 3.23, *p* = 0.0013), confirming the result is not an artifact of this analytic choice. The calculated prediction interval spanned from 7.5% to 25.7%, establishing the upper and lower mathematical boundaries for expected attrition in future experimental replications.

#### 3.5.2. Blood Flow Velocity

**Figure 5:**
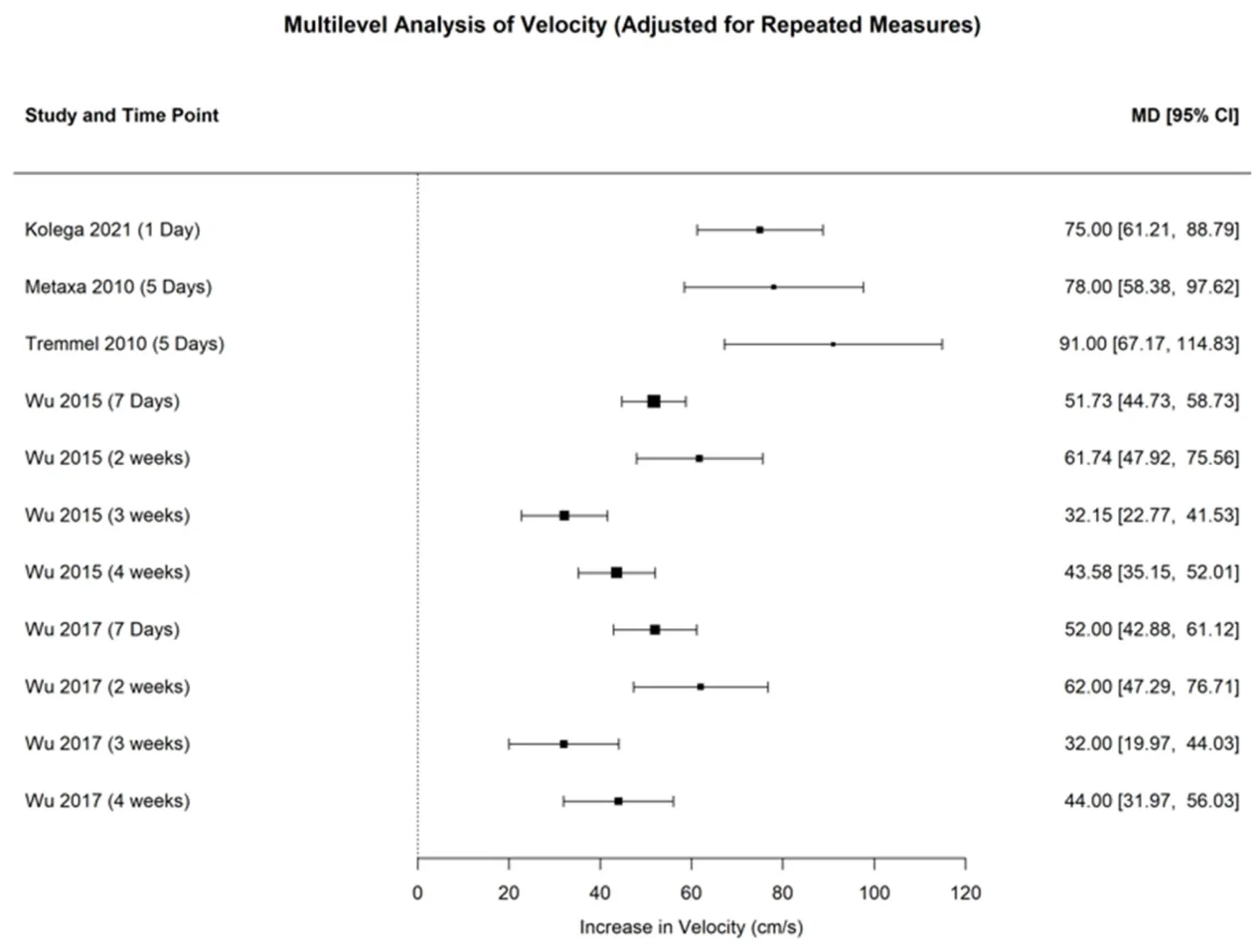
Forest plot illustrating the multilevel analysis of BA blood flow velocity following BCCAL. Each row shows the mean difference (MD, cm/s) between BCCAL and control animals at one study time point. Black squares: MD of each time point (square size reflects the precision of the estimate); horizontal lines: 95% confidence intervals; dotted vertical line at 0: no difference between BCCAL and control animals. Repeated time points were nested within their base study cohort as random-effects components (multilevel mixed-effects meta-analysis); individual time-point estimates are shown here and the pooled estimates are reported in the text. Study labels give the first author, year and follow-up time: Kolega 2021 [15]; Metaxa 2010 [20]; Tremmel 2010 [22]; Wu 2015 [6]; Wu 2017 [23].

To evaluate the dynamic longitudinal alterations in BA blood flow velocity, a Multilevel Mixed-Effects Meta-Analysis was executed (Figure 5). A categorical subgroup analysis revealed a significant, time-dependent elevation in velocity following BCCAL (Omnibus QM = 158.68, df = 2, *p* < 0.0001). In the acute phase (1 to 7 days post-ligation), the diverted cerebral blood flow had a velocity spike, with an MD of 66.57 cm/s higher than sham-operated controls (95% CI: 53.42 to 79.71, *p* < 0.0001), whereas, in the subacute and chronic phases, this phenomenon dampened, narrowing to an MD of 45.33 cm/s above baseline controls (95% CI: 33.88 to 56.78, *p* < 0.0001). This prompted us to apply continuous multilevel meta-regression to quantify this temporal dampening. The regression model proved highly significant (QM = 10.68, df = 1, *p* = 0.0011), calculating a theoretical initial velocity spike of 73.13 cm/s immediately after the surgical occlusion (*p* < 0.0001) followed by a decrease of 1.33 cm/s per day (95% CI: −2.13 to −0.53, *p* = 0.0011).

A cluster-robust sensitivity analysis (5 independent base-study clusters) confirmed the direction and statistical significance of both the acute-phase estimate (66.57 cm/s, 95% CI: 45.43 to 87.71, *p* = 0.0011) and the temporal decay slope (−1.33 cm/s per day, 95% CI: −2.41 to −0.25, *p* = 0.032), with visibly wider intervals reflecting the small number of independent base studies contributing repeated timepoints. The chronic-phase estimate remained significant (*p* = 0.0028) but had only 1 associated degree of freedom under this approach, limiting confidence in its precision and warranting caution against over-interpreting the model-based *p*-values above.

#### 3.5.3. Blood Flow Rate

**Figure 6:**
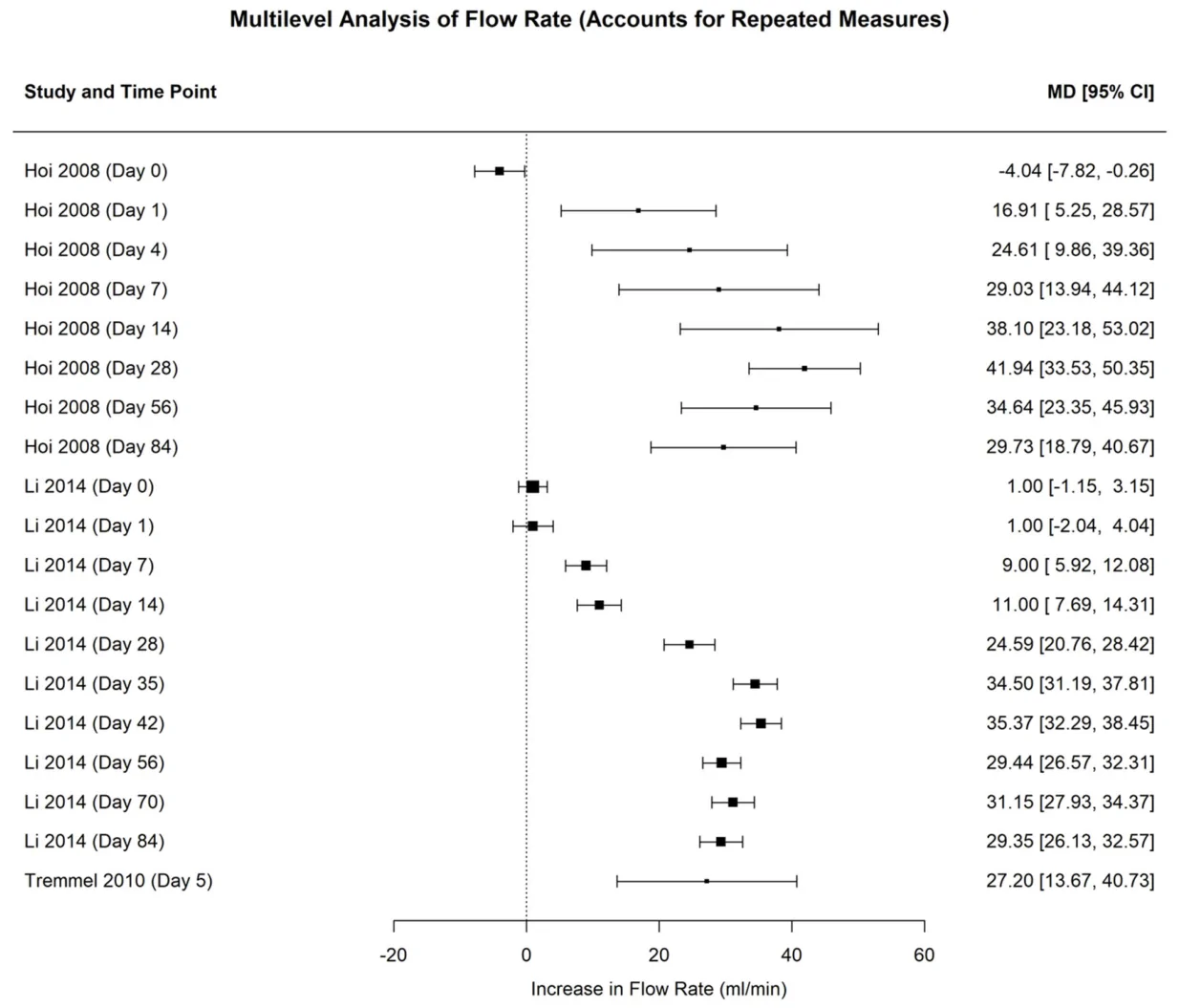
Forest plot illustrating the multilevel analysis of BA volumetric flow rate. Each row shows the mean difference (MD, mL/min) between BCCAL and control animals at one study time point. Black squares: MD of each time point (square size reflects the precision of the estimate); horizontal lines: 95% confidence intervals; dotted vertical line at 0: no difference between BCCAL and control animals. Repeated time points were nested within their base study cohort as random-effects components (multilevel mixed-effects meta-analysis); individual time-point estimates are shown here and the pooled estimates are reported in the text. Study labels give the first author, year and follow-up day: Hoi 2008 [12]; Li 2014 [16]; Tremmel 2010 [22].

To evaluate the progression of volumetric blood flow (mL/min) within the BA while adjusting for repeated longitudinal measurements, a multilevel mixed-effects meta-analysis was conducted (Figure 6). We reduced the statistical inflation that can occur when measuring the same animal cohorts over time by defining the underlying experimental cohorts as a random-effects grouping variable. Because only 3 independent base studies (Hoi 2008 [12], Li 2014 [16], Tremmel 2010 [22]) contribute the 19 timepoint-level effect sizes analyzed here, a cluster-robust variance estimation (RVE) sensitivity analysis, clustered at the base-study level, was additionally performed to verify that the model-based findings below were not an artifact of unmodeled within-study correlation.

A categorical subgroup analysis suggested a stepwise escalation of volumetric flow depending on the surgical phase (Omnibus QM = 67.24, df = 3, *p* < 0.0001). Following bilateral carotid ligation, the acute phase (1 to 7 days) exhibited an immediate volumetric surge, with the basilar flow rate increasing by 16.11 mL/min compared with sham controls (95% CI: 6.08 to 26.15, *p* = 0.0017). However, this acute-phase estimate did not remain statistically significant under the cluster-robust sensitivity analysis (*p* = 0.180, df = 1.78), reflecting the limited number of independent base studies. The chronic-phase estimate (>7 days) was larger, at 33.23 mL/min compared with non-ligated controls (95% CI: 24.06 to 42.39, *p* < 0.0001), and remained statistically significant under the robust check, though only marginally (*p* = 0.043, df = 1.25).

To further characterize this temporal pattern, a continuous multilevel meta-regression was applied to the post-ligation follow-up days (excluding the Day 0 baseline) and was statistically significant under the model-based approach (QM = 7.55, df = 1, *p* = 0.006), estimating an initial post-surgical flow increase of 19.73 mL/min and a positive slope of approximately 0.23 mL/min per day (95% CI: 0.07 to 0.39). This slope did not remain statistically significant under the cluster-robust sensitivity analysis (*p* = 0.266, df = 1.11, 95% CI: −0.88 to 1.33). Given this discrepancy, the temporal escalation in flow rate should be regarded as an observed pattern in the model-based analysis rather than a robustly confirmed finding, pending replication in additional independent cohorts.

#### 3.5.4. BA Vessel Volume

**Figure 7:**
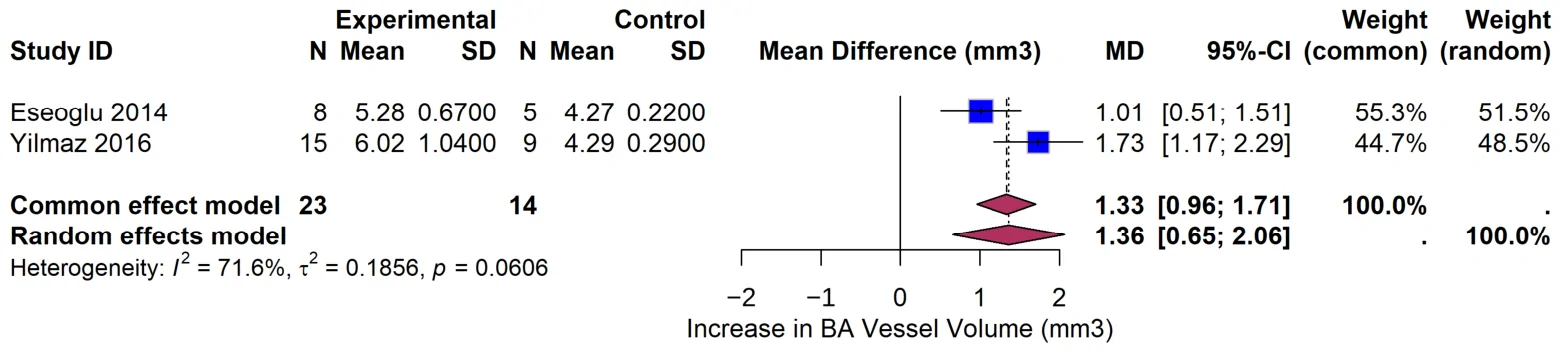
Forest plot for the pairwise meta-analysis of BA vessel volume at 60 days post-ligation. Experimental = BCCAL group; Control = non-ligated animals (control and/or sham); N = number of animals; Mean and SD in mm^3^. Blue squares: mean difference (MD, mm^3^) of each study (square size proportional to its weight); horizontal lines: 95% confidence intervals; maroon diamonds: pooled common-effect and random-effects estimates (diamond width = 95% CI); solid vertical line at 0: no difference between BCCAL and control animals; dotted vertical lines: pooled estimates (1.33 and 1.36 mm^3^). Weights (%) are shown for both models. The random-effects estimate (REML; MD = 1.36 mm^3^, 95% CI 0.65–2.06) is the one reported in the text; with only two studies, I^2^ is not reliably estimable. Study labels give the first author and year: Eseoglu 2014 [10]; Yilmaz 2016 [24].

The dataset comprised two independent studies (*n* = 37 rabbits) that measured the BA volume (mm^3^) using stereological methods at the 60-day post-ligation timepoint, for which we performed a standard continuous pairwise meta-analysis (Figure 7). The two cohorts’ effect estimates differed appreciably; however, with only two studies, the heterogeneity index is not reliably estimable and should be interpreted with care (I^2^ = 71.6%, Q = 3.52, *p* = 0.0606). Under the random-effects framework, the BCCAL procedure induced a highly significant volumetric expansion of the BA compared to non-ligated controls. At 60 days post-ligation, the BAs demonstrated an absolute mean volume increase of 1.36 mm^3^ (95% CI: 0.65 to 2.06). The z-test for overall effect confirmed that this macroscopic structural expansion was statistically significant (z = 3.78, *p* = 0.0002).

#### 3.5.5. Wall Shear Stress

To evaluate the dynamic alterations in WSS (Pa) following BCCAL, a two-step hybrid statistical approach was utilized. First, to assess the consistency of the hemodynamic insult across the two independent laboratories, a standard random-effects pairwise meta-analysis was performed at the 24 h timepoint. The two laboratories’ effect estimates differed significantly, indicating that the magnitude of the acute WSS increase was not fully reproducible across settings (I^2^ = 79.71%, *p* = 0.0264). With only two contributing studies, I^2^ is not a reliable metric here and should be interpreted with caution. A mean WSS increase of 26.06 Pa was found compared to non-ligated controls (95% CI: 13.63 to 38.48, *p* < 0.0001). Second, because longitudinal data spanning Day 1 through 84 were available only from a single study (Hoi 2008 [12]), a within-study regression was performed (QM = 13.60, df = 1, *p* = 0.0002), showing a negative slope. The excess WSS declined at a rate of 0.15 Pa per day (95% CI: −0.23 to −0.07, *p* = 0.0002). As this trend is derived entirely from one contributing study, with no second cohort available for a cluster-based sensitivity analysis, it should be regarded as preliminary and hypothesis-generating rather than a generalizable estimate of the WSS decay rate.

#### 3.5.6. Loss of Internal Elastic Lamina

**Figure 8:**
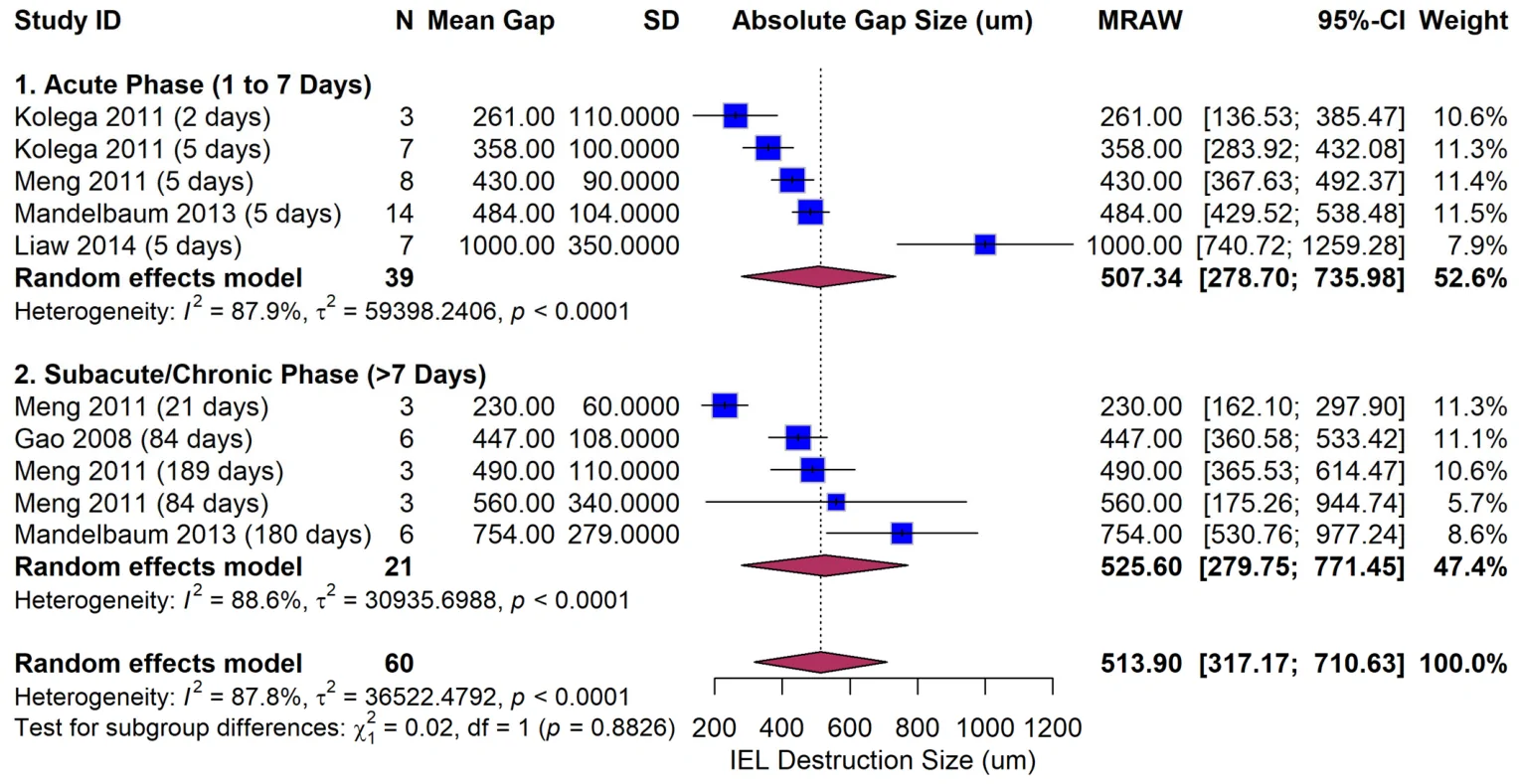
Forest plot of the single-arm continuous meta-analysis detailing absolute IEL destruction gap size (µm) across experimental cohorts. Blue squares: mean IEL gap of each cohort or time point (square size proportional to its weight); horizontal lines: 95% confidence intervals; maroon diamonds: pooled means for each subgroup (acute phase, 1–7 days; subacute/chronic phase, >7 days) and overall, estimated with the multilevel mixed-effects model (repeated time points nested within base studies; diamond width = 95% CI); dotted vertical line: overall pooled mean (513.90 µm). The test for subgroup differences is the omnibus test of moderators. N = number of animals; SD = standard deviation; MRAW = raw (untransformed) mean; I^2^ and τ^2^ = heterogeneity statistics. Study labels give the first author, year and follow-up time: Kolega 2011 [14]; Meng 2011 [19]; Mandelbaum 2013 [18]; Liaw 2014 [17]; Gao 2008 [11].

A multilevel mixed-effects meta-analysis, nesting repeated timepoints within their base study cohort, was performed on the BCCAL experimental cohorts (k = 10 observations from 5 base studies, *n* = 60 animals) to quantify the absolute size of the IEL gap and map its degradation over time (Figure 8). In healthy control rabbits, no structural changes were reported.

The overall model estimated an average IEL destruction gap of 513.90 µm across the lifespan of the BCCAL model (95% CI: 317.17 to 710.63). The acute phase (1 to 7 Days) demonstrated a gap size of 507.34 µm, and the chronic phase (greater than 7 Days) a gap size of 525.60 µm; however, these did not differ significantly (Q = 0.02, *p* = 0.883). A continuous meta-regression using follow-up days as the moderator found a slope of +1.00 µm per day, which was not statistically significant (*p* = 0.106) and did not remain significant under a cluster-robust sensitivity analysis (*p* = 0.186, df = 1.12), consistent with an absence of clear temporal progression in this outcome. Because of high heterogeneity in IEL destruction data (I^2^ = 87.8%), a leave-one-cluster-out sensitivity analysis was conducted, sequentially removing each base study (rather than each individual timepoint, given that several base studies contributed multiple correlated timepoints) and recalculating the pooled estimate. Across all five permutations, the pooled mean gap size ranged from 423.50 µm (with Liaw 2014 [17] removed) to 570.82 µm (with Kolega 2011 [14] removed), remaining within a plausible range around the full-model estimate of 513.90 µm and indicating that no single base study disproportionately drives the overall effect.

#### 3.5.7. Media Thinning

**Figure 9:**
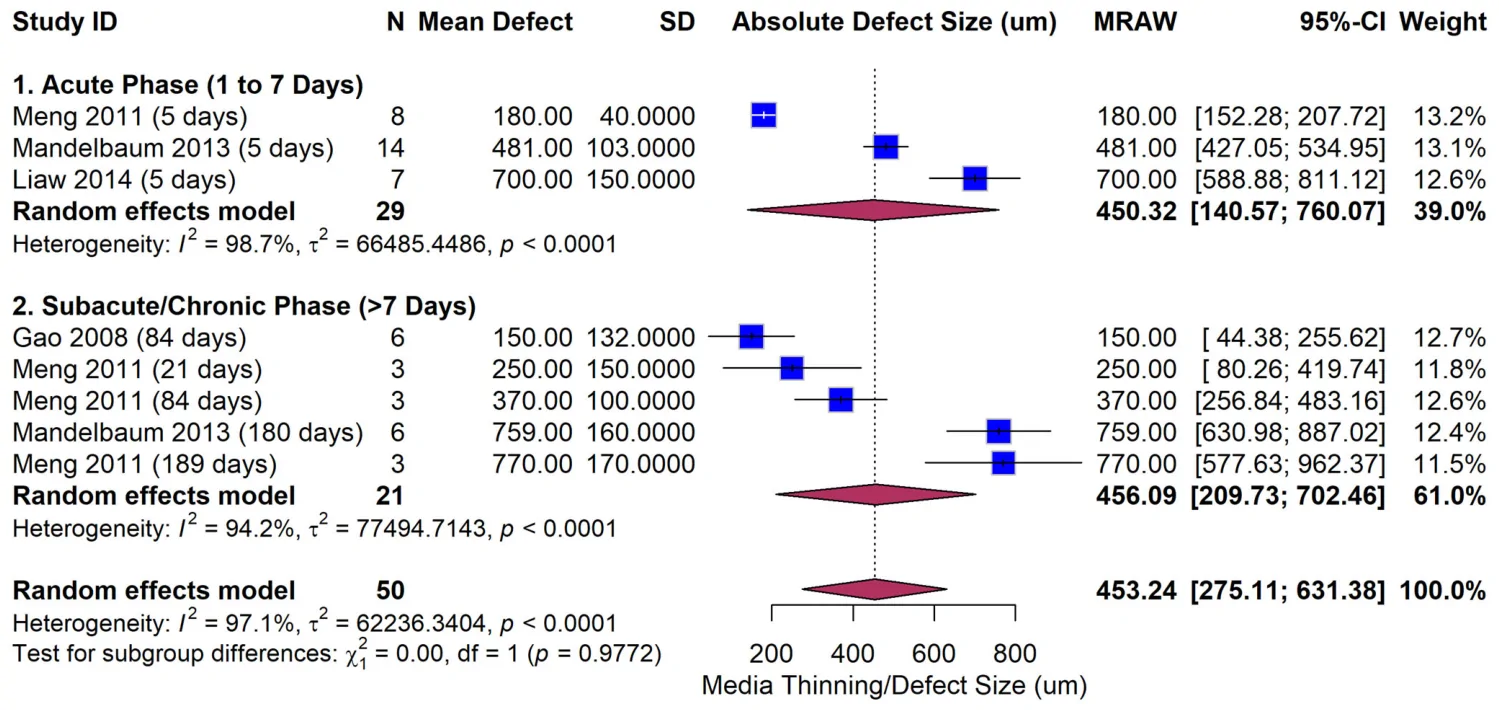
Forest plot of the single-arm continuous meta-analysis detailing absolute media thinning and defect size (µm) across experimental cohorts post-BCCAL. Blue squares: mean media defect of each cohort or time point (square size proportional to its weight); horizontal lines: 95% confidence intervals; maroon diamonds: pooled means for each subgroup (acute phase, 1–7 days; subacute/chronic phase, >7 days) and overall, estimated with the multilevel mixed-effects model (repeated time points nested within base studies; diamond width = 95% CI); dotted vertical line: overall pooled mean (453.24 µm). The test for subgroup differences is the omnibus test of moderators. N = number of animals; SD = standard deviation; MRAW = raw (untransformed) mean; I^2^ and τ^2^ = heterogeneity statistics. Study labels give the first author, year and follow-up time: Gao 2008 [11]; Meng 2011 [19]; Mandelbaum 2013 [18]; Liaw 2014 [17].

A multilevel mixed-effects meta-analysis, nesting repeated timepoints within their base study cohort, was applied to the BCCAL experimental cohorts (k = 8 observations from 4 base studies, *n* = 50 animals) to quantify the media defect (µm) and track its behavior across the experimental timeline (Figure 9). The average medial defect size was estimated at 453.24 µm across the lifespan of the BCCAL model (95% CI: 275.11 to 631.38), unchanged from the unclustered estimate as between-study variance in the overall mean was negligible. The acute phase (1 to 7 Days) registered a medial defect averaging 450.32 µm and the chronic phase (greater than 7 Days) averaged 456.09 µm, with no statistically significant difference between phases (*p* = 0.977). A continuous meta-regression using follow-up days as the predictive moderator yielded a positive slope of +2.22 µm per day. Under the properly clustered model this slope was statistically significant (*p* < 0.0001); however, it did not remain significant under a cluster-robust sensitivity analysis clustered by base study (*p* = 0.193, df = 1.03, 95% CI: −6.28 to 10.71). Given this discrepancy and given that the earlier unclustered analysis had understated the apparent trend rather than correctly rejecting it, the relationship between follow-up time and medial defect size should be regarded as unresolved rather than confidently absent, pending data from additional independent cohorts.

#### 3.5.8. Aneurysm Development Score

**Figure 10:**
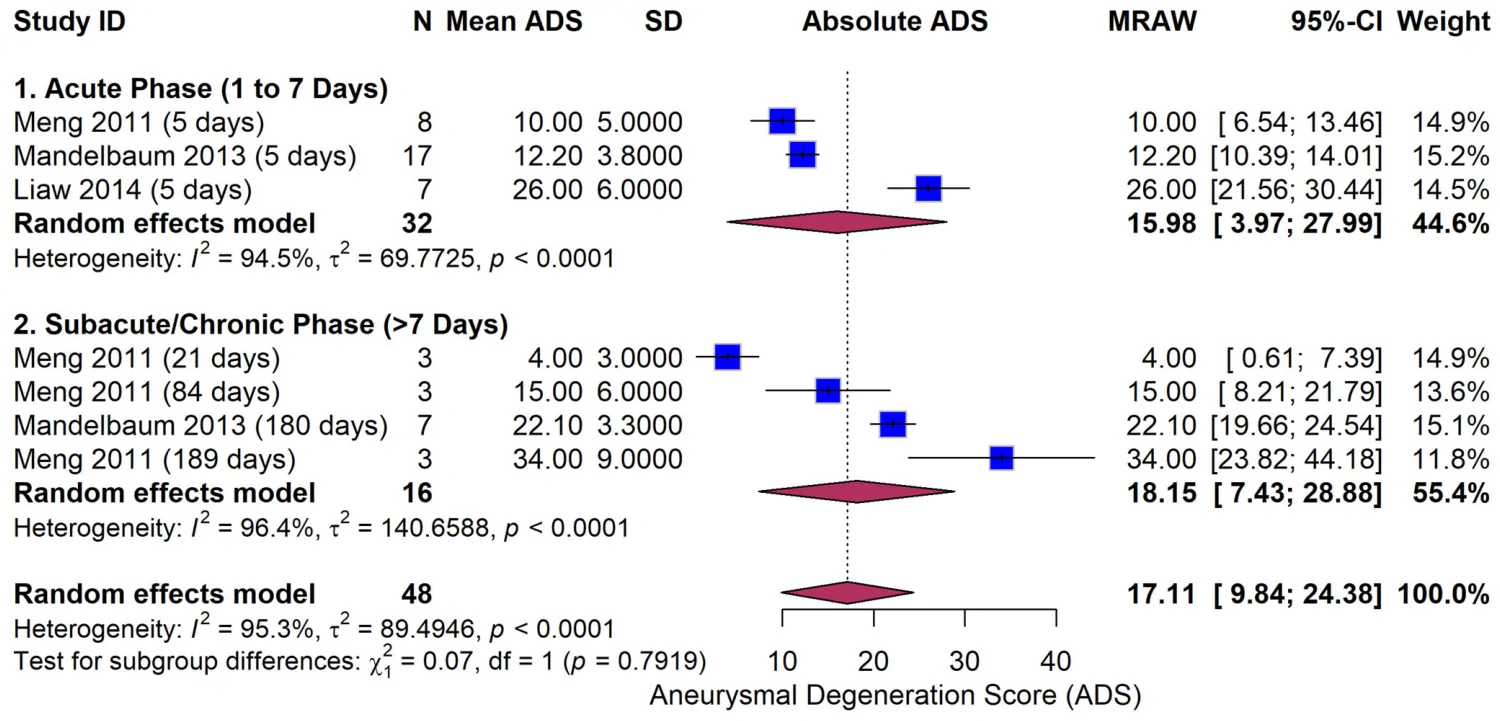
Forest plot of the single-arm continuous meta-analysis evaluating the composite Aneurysm Development Score (ADS) across experimental cohorts post-BCCAL. Blue squares: mean ADS of each cohort or time point (square size proportional to its weight); horizontal lines: 95% confidence intervals; maroon diamonds: pooled mean ADS for each subgroup (acute phase, 1–7 days; subacute/chronic phase, >7 days) and overall, estimated with the multilevel mixed-effects model (repeated time points nested within base studies; diamond width = 95% CI); dotted vertical line: overall pooled mean (17.11 points). The test for subgroup differences is the omnibus test of moderators. N = number of animals; SD = standard deviation; MRAW = raw (untransformed) mean; I^2^ and τ^2^ = heterogeneity statistics. Study labels give the first author, year and follow-up time: Meng 2011 [19]; Mandelbaum 2013 [18]; Liaw 2014 [17].

A multilevel mixed-effects meta-analysis, nesting repeated timepoints within their base study cohort, was applied to BCCAL experimental cohorts (k = 7 observations from 3 base studies, *n* = 48 animals) to quantify the composite severity of the tissue degeneration and evaluate its progression over time (Figure 10).

The overall model estimated an average ADS of 17.11 points across the BCCAL model’s aggregate lifespan (95% CI: 9.84 to 24.38), unchanged from the unclustered estimate. Pooled scores were 15.98 points in the acute phase (1 to 7 days) and 18.15 points in the chronic phase (>7 days), with no statistically significant difference between phases (*p* = 0.792). The model intercept indicated an immediate post-surgical tissue degeneration score of 13.21 points (*p* = 0.016), with a positive regression slope of +0.089 points per day. This slope was statistically significant under the properly clustered model (*p* = 0.0065) but did not remain significant under a cluster-robust sensitivity analysis (*p* = 0.281, df = 1.09, 95% CI: −0.38 to 0.56). As with Media Thinning, this discrepancy indicates the temporal relationship should be treated as unresolved rather than ruled out, given only 3 independent base studies contribute to this outcome.

### 3.6. Narrative Synthesis of Secondary Morphological and Hemodynamic Parameters

Due to high heterogeneity and insufficient absolute values reported, we have evaluated the BA diameter, VDI and WSS gradient through a structured narrative synthesis to provide contextual support for the primary meta-analysis.

Following BCCAL, BA undergoes rapid morphological adaptation characterized by an increase in luminal diameter. Hoi et al. demonstrated that BA diameter increased significantly after bilateral ligation and that vessel caliber gradually increased, extending beyond 28 days [12]. These findings were corroborated by Tutino et al., who reported a 54% augmentation at just 5 days post-ligation compared to sham-operated controls [5]. Gao et al. have also noted an increase in BA diameter, with basilar flow rate increasing by up to 900% [11]. Therefore, BA enlargement occurs after BCCAL, and the BA initially undergoes vasodilatation that is later maintained to accommodate the increase in basilar flow.

Regarding the WSS gradients, Kolega et al. noted that they are concentrated in the regions where the most severe destructive changes to the vessel wall are observed [14,15]. The effect was demonstrated by Metaxa et al., who reported positive WSS gradients at the bifurcation apex. They found a fivefold increase compared to pre-ligation baselines and a distinct distribution correlated with localized tissue destruction. In fact, their model indicated that when WSS exceeded 122 Pa, and the positive WSSG exceeded 530 Pa/mm, destructive structural remodeling occurred 100% of the time [20].

VDI is a histomorphological parameter used to evaluate the severity of arterial dilatation and vasospasm. To quantify this structural change, Aygul et al. presented the VDI using the formula VDI = (R2 − r2)/r2, which calculates the ratio of the wall-ring surface area to the lumen surface area, where R represents the external radius and r represents the internal luminal radius. Across the posterior cerebral circulation, BCCAL consistently induces structural changes that decrease VDI values. In the posterior communicating artery, Aygul et al. observed that a normal baseline VDI of 1.5 ± 0.440 fell to between 1.033 ± 0.108 and 0.875 ± 0.132 in ligated animal cohorts [9]. Yilmaz et al. applied this index to the vertebral arteries, recording a significant decrease in VDI from 2.30 ± 0.91 in controls down to 1.04 ± 0.13 in animals exhibiting pronounced vasodilatation [24]. This structural distortion is similarly found in the BA. Kilic et al. reported a reduction in VDI from 1.65 ± 0.12 in control subjects to 0.976 ± 0.112 following ligation [13]. Aydin et al. further corroborated this BA yielding, reporting that the baseline VDI of 1.390 ± 0.220 decreased to 0.019 ± 0.011 under maximum hemodynamic stress [8]. These results indicate that, following BCCAL, the vessel wall thins in response to lumen expansion throughout the posterior circulation.

## 4. Discussion

Our systematic review and meta-analysis highlight the value of BCCAL in inducing IAs in rabbits. Although other models for inducing/creating aneurysm-like lesions, such as venous graft aneurysms or elastase-induced carotid aneurysms, have been developed, the model we are investigating in this review replicates the pathophysiological mechanism of IA development [2]. Initially described by Hassler in 1963, by ligating both common carotid arteries, the model induces aneurysms of the posterior circulation via hemodynamic stress [4]. The main findings of our systematic meta-analysis show that after ligation, there is an immediate increase in blood flow velocity and rate, which induces a frictional environment and a compensatory vasodilation in the posterior circulation. From a histopathological perspective, this hemodynamic change is proposed as the primary destructive driver, plausibly activating inflammatory pathways and enzymatic degradation of the IEL and media. However, this causal sequence was not directly tested within the present meta-analysis and is inferred by synthesizing findings across the individually included studies. To our knowledge, this is the first study to evaluate the robustness and reproducibility of this model. We found that aneurysm induction is highly dependent on what the researcher is looking for. Only 3 studies reported macroscopic aneurysm formation at the level of the Circle of Willis, yielding an incidence rate of 23.4% [6,8,21]. This result aligns with reports from other experimental models. For instance, Tada et al. reported an equivalent 23% incidence of definitive saccular aneurysms at the 12-week timepoint at the level of the left anterior cerebral artery-olfactory artery in rats [26]. This consistency reinforces the notion that mature aneurysm formation is a challenging endpoint to achieve.

However, if the main focus is destructive aneurysmal remodeling rather than a mature macroscopic aneurysm, some evidence of localized structural injury was documented across all seven studies reporting this outcome (60 of 60 animals). This apparent consistency should be read with caution: less than half of these studies confirmed a visible macroscopic bulge, while the remainder relied on histological or molecular criteria alone, so the 100% figure reflects agreement that some form of destructive change occurs, not that a single, comparable lesion was observed in every laboratory. Within that caveat, the BCCAL model reliably induces detectable aneurysm-like structural injury and serves as a platform for investigating the early stages of aneurysmal wall damage.

Despite its efficacy in inducing localized aneurysmal remodeling, the BCCAL model exacts a systemic toll on the subjects. Complications such as subarachnoid hemorrhage, ischemic attacks, convulsions, fever, cardiac arrhythmia and breathing disturbances were observed after ligation [8,9,10,13,24,25]. The mortality rate of 16.6%, with a consistently low heterogeneity estimate across laboratories, suggests that this attrition is an inherent property of the BCCAL model rather than an artifact of inconsistent laboratory environments or poor surgical skill. However, the width of the I^2^ confidence interval means inter-laboratory or procedural effects cannot be fully excluded.

For investigators planning future preclinical trials, these findings offer practical utility for experimental design. Utilizing the 23.4% macroscopic aneurysm incidence rate alongside the observed 16.6% mortality rate establishes a preliminary baseline for power analyses, enabling more precise sample size calculations and a priori estimates of study power. However, this baseline should be interpreted in light of the moderate-to-low quality of the underlying evidence and the small number of studies (k = 3) contributing to the aneurysm incidence estimate.

Our findings suggest that after BCCAL, there is an initial acute change in the hemodynamics of the posterior circulation, characterized by an immediate increase in blood flow velocity of 66.57 cm/s, blood flow rate of 16.11 mL/min (a model-based estimate that did not remain statistically significant under cluster-robust sensitivity analysis; see Results) and WSS of 26.06 Pa compared to controls. This phenomenon occurs as cardiac output is redirected into the posterior circulation to prevent brain ischemia. These findings induce frictional abrasive forces and mechanical stress upon the endothelium, provoking enzymatic degradation of the IEL and medial defect. In both 2- and 5-day ligated rabbits, MMP-9 was reported elevated and localized in the region of IEL disruption. Similarly, the MMP-2 expression was increased in the periapical areas with IEL destruction [14]. The effect is almost immediate within the first 7 days, with a pooled IEL destruction gap size of 507.34 µm and a media defect of 450.32 µm. This alteration closely mimics the pathophysiology of acute hypertension-mediated vascular injury in humans, with an increase in resistance and further endothelial damage [27]. Moreover, this histopathological damage is strictly focal. As demonstrated by Metaxa et al., structural degradation preferentially occurs at the apex of the BA (a region subjected to peak WSS and a 5-fold surge in the WSS gradient). Conversely, straight arterial segments subjected to elevated, yet uniform, WSS safely undergo adaptive luminal enlargement without structural failure [20]. However, inflammatory markers are present throughout the basilar bifurcation, with eNOS staining in the intima, activated NF-κB and MCP-1 staining at the basilar terminus in ligated animals, and increased KLF2 protein expression compared to sham [17,18,23]. Interestingly, classical inflammatory cellular infiltration does not appear to drive this initial damage, as neutrophils, T lymphocytes and macrophages were rare in the periapical region of both control and ligated animals [14].

Following the acute hemodynamic insult, the posterior circulation arteries undergo continuous vascular remodeling and vasodilatation. This phase is characterized by a daily decrease in basilar blood flow velocity of 1.33 cm/s and a corresponding WSS decay rate of 0.15 Pa/day. The latter is derived from within-study regression in a single contributing cohort (Hoi 2008 [12]) and therefore preliminary pending confirmation in additional studies. Simultaneously, the model-based estimate suggests the persistent demand for cerebral perfusion may accommodate an additional 0.23 mL/min of blood flow per day, though this temporal trend did not remain statistically significant under cluster-robust sensitivity analysis and should be interpreted as preliminary. The compensatory vasodilatation response is quantified using the VDI, a calculated ratio of wall-ring to lumen surface area. The principle being, as a blood vessel dilates and its wall thins to accommodate increased blood flow, the internal radius approaches the external radius, resulting in a significantly lower VDI value. Across all experiments that reported it, BCCAL consistently produced a marked reduction in VDI relative to non-ligated controls in all vessels of the posterior circulation, demonstrating that structural vasodilatation and luminal enlargement occur after BCCAL [8,9,13,24].

Given a mean follow-up duration of 90.88 days across the included studies (*n* = 17), we evaluated the chronic hemodynamic adaptations within this model. As the BA undergoes geometric dilatation to attenuate localized wall stress, regional vascular resistance declines. This structural adaptation results in a decreased blood flow velocity (mean 45.33 cm/s throughout the chronic phase) and a normalization of WSS within an expanded total vessel volume of 1.36 mm^3^ at 60 days. Concurrently, the posterior circulation entirely assumes the cerebral arterial supply, demonstrating a twofold increase in volumetric blood flow (33.23 mL/min) compared to non-ligated controls. Quantitatively, this outward geometric remodeling is corroborated by an increase in BA diameter that reaches up to 1.37 mm by day 84 post-ligation [12]. Although these parameters exhibit a trend toward stabilization, the posterior vasculature remains subjected to persistently elevated mechanical stress to maintain this compensatory, albeit abnormal, hemodynamic environment. Macroscopically, the physical toll is evident, with vessels showing elongation and dolichoectasia, manifesting as an elevated tortuosity index throughout the chronic phase [7,10,12]. Consequently, this model may parallel aspects of the chronic hemodynamic burden seen in systemic hypertension, in that the vasculature is subjected to sustained, elevated mechanical loads. This, in turn, triggers systemic endothelial dysfunction, which is considered a primary risk factor for IAs [28,29].

Perhaps the most unexpected finding from the histopathological analyses is the absence of a statistically detectable difference in tissue damage between the acute and chronic phases. Aneurysmal pathology is often conceptualized as a slow, progressive degenerative process [30]. Our data suggests that, in the BCCAL model, the initial mechanical insult may not be followed by substantial further structural progression, though the absence of a significant subgroup difference should not be read as proof of biological equivalence between phases. The compensatory vasodilatation mechanism may reduce the mechanical trigger for ongoing inflammation and enzymatic degradation, consistent with the IEL pooled gap size remaining broadly similar between phases (507.34 µm acute vs. 525.60 µm chronic) and the media thinning likewise (450.32 µm acute vs. 456.09 µm chronic). This raises the possibility that the initial structural defect does not substantially expand over the observed follow-up period, though fatal rupture remains a distinct risk if the acute hemodynamic stress forces the vessel past its upper rupture limit [8,20]. Also, this raises the question whether the model is suitable for studying the chronic, multi-year progression of human IAs or whether its value lies in providing a highly stable structural defect for researching pharmacological agents designed to stabilize and treat IAs. While this systematic review and meta-analysis provide the first comprehensive evaluation of the rabbit BCCAL model, several limitations must be acknowledged, stemming from both our review methodology and the inherent nature of the primary literature. Regarding the search strategy, our inclusion criteria were strictly limited to peer-reviewed, published articles. The deliberate exclusion of gray literature, specifically conference papers (*n* = 4), combined with the exclusion of foreign language publications (*n* = 3), introduces a risk of language and publication bias. Consequently, the literature pool may overrepresent experiments published in English-language journals, while potentially overlooking null results or methodological variations presented in regional or unpublished forums.

A critical limitation of the primary literature is the pervasive lack of experimental transparency, as evidenced by our ARRIVE and SYRCLE assessments. A deficit in basic experimental rigor was observed: no study reported an a priori sample size calculation to mathematically justify their animal cohorts, and only 13.6% explicitly detailed their randomization sequence generation. Furthermore, the handling of procedural mortality introduces a significant risk of attrition bias. Because the BCCAL model induces an acute hemodynamic shift, several studies reported early animal deaths. However, these subjects were frequently excluded from the final denominator rather than being accounted for analytically (e.g., via intent-to-treat analysis). This selective exclusion may artificially inflate both overall survival rates and the perceived incidence of macroscopic aneurysm formation in surviving cohorts.

Due to fractured and inconsistent reporting styles across different laboratories, several critical secondary outcomes could not be mathematically pooled. Structural and hemodynamic parameters, such as VDI, specific BA diameter measurements, and spatial WSS gradients, were reported sparsely. Consequently, these metrics had to be relegated to a narrative synthesis rather than a rigorous quantitative meta-analysis, which limited our ability to generate precise, pooled numerical effect sizes for these variables.

Finally, the biological mechanics of the rabbit BCCAL model itself present distinct translational limitations. While this model is suitable for studying wall-healing pharmacological agents, it fundamentally fails to replicate the chronic, multi-year progressive growth and rupture cycles characteristic of human IAs. Furthermore, the 16.6% surgical mortality rate introduces a notable baseline attrition factor, posing a practical logistical limitation that requires larger initial animal cohorts to ensure adequately powered experimental endpoints.

### Translational Framework for Experimental Design

Beyond characterizing the biological behavior of the BCCAL model, these pooled estimates carry practical value for investigators designing future preclinical neurovascular studies. We propose the following framework as preliminary planning guidance rather than a validated protocol, given the limited number of contributing studies for several outcomes (k = 3 for macroscopic aneurysm formation, k = 7 for destructive remodeling) and the moderate-to-low overall quality of the underlying evidence discussed above.

Sample-size planning. The pooled macroscopic aneurysm incidence of 23.4% (97.5% CI: 14.3–35.8%) can serve as a baseline event-rate input for two-proportion power calculations. For example, a study designed to detect a doubling of this incidence (to approximately 47%) following a candidate pharmacological or surgical modification, using a two-sided test with α = 0.025 (consistent with the multiplicity correction applied to our co-primary outcomes) and 80% power, would require approximately 45–50 animals per arm under standard proportion-comparison formulae. Because the underlying incidence estimate itself carries considerable uncertainty, investigators are encouraged to repeat this calculation using the bounds of the reported confidence interval rather than the point estimate alone, to obtain a plausible range for required sample size. Independently of this calculation, the observed 16.6% absolute mortality increase associated with BCCAL (95% CI: 9.35–23.90%) should be factored into recruitment. The number of animals recruited into a study should exceed the analytically required sample size by a margin sufficient to absorb expected procedural attrition, with both the “enrolled N” and the “expected analyzable N” reported transparently in the study protocol.

Timing of intervention and assessment. Our temporal analysis identifies two biologically distinct windows that carry different implications for study design (Table 3). The acute phase (1–7 days post-ligation) is characterized by the peak hemodynamic insult (blood flow velocity +66.57 cm/s and WSS +26.06 Pa above sham controls) and the structural injury (IEL destruction gap 507.34 µm; media thinning 450.32 µm). This window is therefore best suited to studies targeting the initiating mechanical and inflammatory mechanisms of aneurysm formation, such as investigations of MMP activity, endothelial dysfunction, or early enzymatic degradation pathways. The chronic phase (>7 days) is instead characterized by compensatory vasodilatation, a moderation of blood flow velocity (MD 45.33 cm/s above baseline), a doubling of volumetric flow (33.23 mL/min), and measurable basilar artery volumetric expansion (+1.36 mm^3^ by 60 days). Because our analysis could not conclusively determine whether the structural defect remains stable or progresses further beyond this point, studies aiming to confirm mature aneurysm formation should extend follow-up substantially.

Endpoint selection. We propose that endpoint selection be guided explicitly by the research question rather than defaulting to a single fixed protocol (Table 4). Studies interrogating the initiating mechanism of aneurysmal injury are best served by hemodynamic imaging (Doppler-derived velocity and WSS) paired with early histological assessment of the IEL and tunica media. Studies aiming to confirm aneurysm induction efficacy of a modified surgical or pharmacological protocol should prioritize macroscopic inspection at the basilar terminus/Circle of Willis at a late timepoint, given that this is the only endpoint category with a directly comparable pooled incidence in the current literature. Studies testing candidate stabilizing or preventive therapies may benefit from a paired design spanning both phases, using acute hemodynamic and histological endpoints to confirm target engagement and a late macroscopic endpoint to assess whether the compensatory vasodilatation response observed in this analysis is sufficient, in combination with the intervention, to arrest further structural change.

Proposed decision sequence. We summarize the above considerations as a simple sequential framework: (1) define whether the research question is mechanistic (acute injury pathways) or outcome-confirmatory (aneurysm induction efficacy); (2) select the corresponding assessment window from Table 3; (3) select the corresponding endpoint(s) from Table 4; and (4) power the study using the worked example above, explicitly stating the assumed baseline event rate, expected effect size, and anticipated attrition.

These recommendations should be interpreted alongside the limitations discussed above, in particular, the small number of studies contributing to the incidence and mortality estimates, and the absence of a priori power calculations across the entire body of primary literature we reviewed. They are intended to provide a transparent, revisable starting point for experimental planning rather than a fixed standard.

## 5. Conclusions

This systematic review and meta-analysis indicate that the rabbit BCCAL model reliably induces early-stage aneurysmal remodeling, with evidence of structural injury reported across all animals examined for this outcome, though the operational definition of “remodeling” varied across contributing studies. Histopathologically, the model appears to operate via a rapid, acute mechanical insult that provokes enzymatic degradation within the vessel walls of the posterior circulation, followed by a compensatory vasodilation reaction that may attenuate the initial hemodynamic injury. However, whether the initial structural damage remains static or progresses further over time could not be conclusively determined from the available data. Beyond these biological findings, our review also indicates that the current literature is substantially limited by a lack of methodological transparency, particularly concerning a priori sample size calculations and the improper analytical handling of procedural mortality. For investigators designing future preclinical trials who are interested in macroscopic aneurysm induction, study power calculations may be informed by the observed aneurysm incidence rate of 23.4% and the observed 16.6% baseline surgical mortality rate. Ultimately, the true translational value of this model lies in its utility for investigating inflammatory markers and testing targeted pharmacological therapies aimed at aneurysm prevention and stabilization.

## Figures and Tables

**Table 2:** Operational definitions of destructive aneurysmal remodeling across the seven contributing studies, illustrating the heterogeneity underlying the pooled 100% incidence estimate.

Study	Events	Timepoint	What Was Assessed
Gao 2008 [11]	6/6	12 weeks	Macroscopic bulge, graded via a “Nascent Aneurysm Index”
Hassler 1963 [4]	2/2	1–5 months	Macroscopic bulging medial defect
Kolega 2011 [14]	10/10	2–5 days	Localized histological destructive changes
Li 2014 [16]	10/10	3 months	Histological/molecular markers (endothelial cell apoptosis, loss of β-catenin expression)
Liaw 2014 [17]	7/7	5 days	IEL loss, media thinning, bulge formation and elevated ADS
Meng 2011 [19]	17/17	27 weeks	Morphology “indicative of” destructive remodeling—no precise criterion stated
Metaxa 2010 [20]	8/8	5 days	IEL damage mapped by location relative to WSS gradient

**Table 3:** Proposed assessment windows for BCCAL experimental design, based on pooled temporal findings from this meta-analysis.

Phase	Post-Ligation Window	Hemodynamic Signal	Structural Signal	Interpretation
Acute	1–7 days	Blood flow velocity: MD +66.57 cm/s vs. sham; WSS: MD +26.06 Pa vs. sham	IEL destruction gap: 507.34 µm; media thinning: 450.32 µm; ADS ≈ 16 points	Peak mechanical/inflammatory insult window appropriate for mechanistic studies of injury initiation
Chronic	>7 days (mean follow-up 90.88 days)	Blood flow velocity moderates to MD +45.33 cm/s vs. sham; volumetric flow: MD +33.23 mL/min vs. sham; BA volume: +1.36 mm^3^ at 60 days	IEL/media/ADS values remain similar to acute phase	Compensatory/Vasodilatory window appropriate for outcome-confirmatory studies and for testing whether an intervention alters the trajectory of an already-established defect

**Table 4:** Proposed endpoint selection by research question for BCCAL experimental design.

Research Question	Recommended Window	Recommended Endpoint(s)
Mechanistic—injury initiation (e.g., MMP activity, endothelial dysfunction, enzymatic IEL/media degradation)	Acute, 1–7 days post-ligation	Doppler-derived blood flow velocity and WSS; histological IEL gap size and media thickness
Outcome confirmatory—aneurysm induction efficacy of a modified surgical/pharmacological protocol	Late chronic, >8 weeks (ideally minimum 90 days matching the mean follow-up of comparable studies)	Macroscopic inspection at the basilar terminus/Circle of Willis
Therapeutic/preventive—testing a candidate stabilizing or preventive agent	Paired: acute + late chronic	Acute hemodynamic and histological endpoints (target engagement) plus late macroscopic endpoint (structural outcome)

## Data Availability

The original contributions presented in this study are included in the article/Supplementary Material. Further inquiries can be directed to the corresponding author.

## Supplementary Materials

The following supporting information can be downloaded at: https://www.mdpi.com/article/10.3390/medsci14060645/s1, Supplementary_Material.pdf: Search Strategy with Results until 5 September 2026; Supplementary_Material_S2.pdf, Table S1: Study and Outcome Contribution Matrix, indicating which of the 22 included studies contributed data to each quantitative endpoint; Supplementary_Material_S3.pdf, PRISMA 2020 Checklist [31]: completed checklist indicating the manuscript section and line number where each PRISMA 2020 reporting item is addressed.

## Abbreviations

The following abbreviations are used in this manuscript:

ADS	Aneurysm Development Score
ARRIVE	Animals in Research: Reporting in Vivo Experiments
BA	Basilar Artery
BCCAL	Bilateral Common Carotid Artery Ligation
CI	Confidence Interval
eNOS	Endothelial Nitric Oxide Synthase
GLMM	Generalized Linear Mixed Model
IA(s)	Intracranial Aneurysm(s)
IEL	Internal Elastic Lamina
i.m./IM	Intramuscular
KLF2	Kruppel-like Factor 2
MCP-1	Monocyte Chemoattractant Protein-1
MD	Mean Difference
ML	Maximum Likelihood
MMP-2	Matrix Metalloproteinase-2
MMP-9	Matrix Metalloproteinase-9
NF-κB	Nuclear Factor kappa B
NR	Not Reported
NZW	New Zealand White
PRISMA	Preferred Reporting Items for Systematic Reviews and Meta-Analyses
PROSPERO	International Prospective Register of Systematic Reviews
RD	Risk Difference
REML	Restricted Maximum-Likelihood
RVE	Cluster-robust variance estimation
s.c.	Subcutaneous
SYRCLE	Systematic Review Centre of Laboratory Animal Experimentation
VDI	Vasodilatation Index
WSS	Wall Shear Stress
WSSG	Wall Shear Stress Gradient
