# Supplementary material for: Hemodynamic Induction of Intracranial Aneurysms via Bilateral Common Carotid Artery Ligation in Rabbits: A Systematic Review and Meta-Analysis
Source: Med Sci (Basel). 2026 Oct 7;14(6):645. doi: 10.3390/medsci14060645 (PMC13706827; doi:10.3390/medsci14060645)
Supplement: Supplementary file 1 [file medsci-14-00645-s001.zip › Supplementary_Material.pdf]

Supplementary Material
Search Strategy with Results until 5th September 2026

PubMed		
#1	"Rabbits"[Mesh] OR "Rabbits"[tw] OR "Rabbit"[tw]	409390
#2	"Carotid Arteries"[Mesh] OR "Ligation"[Mesh] OR "Carotid Artery Ligation"[tw] OR "Carotid Ligation"[tw] OR "BCCAL"[tw] OR "Bilateral Common Carotid Artery Ligation"[tw] OR "Bilaterally Common Carotid Artery Ligation"[tw] OR "Carotid occlusion"[tw] OR "Flow manipulation"[tw] OR "Flow increase"[tw] OR "Flow velocity"[tw]	163840
#3	#1 AND #2	4646
#4	"Intracranial Aneurysm"[Mesh] OR "Aneurysm*"[tw] OR "Basilar Artery"[tw] OR "Basilar Terminus"[tw] OR "Basilar Arteries"[tw] OR "Vertebral Artery"[tw] OR "Vertebrobasilar"[tw] OR "Vascular Remodeling"[Mesh] OR "Vasculature"[tw] OR "Remodel*"[tw] OR "Reconfiguration"[tw] OR "Wall Shear Stress"[tw] OR "WSS"[tw] OR "Hemodynamic*"[tw] OR "Vessel Diameter"[tw] OR "Wall Thickness"[tw] OR "Media Thickness"[tw] OR "Morphology"[tw] OR "Histopathology"[tw] OR "Morphohistopathological"[tw] OR "Internal Elastic Lamina"[tw] OR "IEL"[tw] OR "Aneurysmal Damage Score"[tw]	1406260
#5	#3 AND #4	990

Embase		
#1	rabbit'/exp OR 'rabbit':ti,ab OR 'rabbits':ti,ab	424399
#2	carotid artery'/exp OR 'artery ligation'/exp OR 'carotid artery ligation':ti,ab OR 'carotid ligation':ti,ab OR 'bccal':ti,ab OR 'bilateral common carotid artery ligation':ti,ab OR 'bilaterally common carotid artery ligation':ti,ab OR 'carotid occlusion':ti,ab OR 'flow manipulation':ti,ab OR 'flow increase':ti,ab OR 'flow velocity':ti,ab	248608
#3	#1 AND #2	4388
#4	intracranial aneurysm'/exp OR 'aneurysm*':ti,ab OR 'basilar artery':ti,ab OR 'basilar terminus':ti,ab OR 'basilar arteries':ti,ab OR 'vertebral artery':ti,ab OR 'vertebrobasilar':ti,ab OR 'vascular remodeling'/exp OR 'vasculature':ti,ab OR 'remodel*':ti,ab OR 'reconfiguration':ti,ab OR 'wall shear stress':ti,ab OR 'wss':ti,ab OR 'hemodynamic*':ti,ab OR 'vessel diameter':ti,ab OR 'wall thickness':ti,ab OR 'media thickness':ti,ab OR 'morphology':ti,ab OR 'histopathology':ti,ab OR 'morphohistopathological':ti,ab OR 'internal elastic lamina':ti,ab OR 'iel':ti,ab OR 'aneurysmal damage score':ti,ab	1697958
#5	#3 AND #4	1089

WoS		
#1	TS=("rabbit" OR "rabbits")	693,782
#2	TS=("carotid artery ligation" OR "carotid ligation" OR "BCCAL" OR "bilateral common carotid artery ligation" OR "bilaterally common carotid artery ligation" OR "carotid occlusion" OR "flow manipulation" OR "flow increase" OR "flow velocity" OR "carotid arter*" OR "ligation")	501127
#3	#1 AND #2	10590
#4	TS=("aneurysm*" OR "basilar artery" OR "basilar terminus" OR "basilar arteries" OR "vertebral artery" OR "vertebrobasilar" OR "vascular remodel*" OR "vasculature" OR "remodel*" OR "reconfiguration" OR "wall shear stress" OR "WSS" OR "hemodynamic*" OR "vessel diameter" OR "wall thickness" OR "media thickness" OR "morphology" OR "histopathology" OR "morphohistopathological" OR "internal elastic lamina" OR "IEL" OR "aneurysmal damage score")	7336909
#5	#3 AND #4	2881
