# Supplementary material for: Hemodynamic Induction of Intracranial Aneurysms via Bilateral Common Carotid Artery Ligation in Rabbits: A Systematic Review and Meta-Analysis
Source: Med Sci (Basel). 2026 Oct 7;14(6):645. doi: 10.3390/medsci14060645 (PMC13706827; doi:10.3390/medsci14060645)
Supplement: Supplementary file 1 [file medsci-14-00645-s001.zip › Supplementary_Material_S2.pdf]

***Table S1.*** *Study and outcome contribution matrix. Checkmarks indicate which of the 22 included studies contributed quantitative data to each pooled outcome (Mortality, Macroscopic Aneurysm Formation, Destructive Aneurysmal Remodeling, Blood Flow Velocity, Wall Shear Stress, Flow Rate, Basilar Artery Volume, Internal Elastic Lamina [IEL] Loss, Media Thinning, and Aneurysmal Development Score [ADS]). Two included studies ( Tutino 2013 and Fumoto 2021) are shown with no checkmarks, as they contributed only to the narrative synthesis of basilar artery diameter and tortuosity index findings, respectively, and did not contribute to any pooled quantitative analysis.*

| **Study [ref]** | **Mortality** | **Outcome A (Macro. Aneurysm)** | **Outcome B (Destructive Remodel.)** | **Velocity** | **WSS** | **Flow Rate** | **BA Volume** | **IEL Loss** | **Media Thinning** | **ADS** |
|---|---|---|---|---|---|---|---|---|---|---|
| Aydin 2020 [20] | ✓ | ✓ | | | | | | | | |
| Aygul 2013 [17] | ✓ | | | | | | | | | |
| Eseoglu 2014 [21] | ✓ | | | | | | ✓ | | | |
| Fumoto 2021 [7] | | | | | | | | | | |
| Gao 2008 [8] | | | ✓ | | | | | ✓ | ✓ | |
| Hassler 1963 [4] | | | ✓ | | | | | | | |
| Hoi 2008 [14] | | | | | ✓ | ✓ | | | | |
| Kilic 2019 [19] | ✓ | | | | | | | | | |
| Kolega 2011 [10] | | | ✓ | | | | | ✓ | | |
| Kolega 2021 [16] | | | | ✓ | ✓ | | | | | |
| Li 2014 [11] | | | ✓ | | | ✓ | | | | |
| Liaw 2014 [9] | | | ✓ | | | | | ✓ | ✓ | ✓ |
| Mandelbaum 2013 [22] | | | | | | | | ✓ | ✓ | ✓ |
| Meng 2011 [12] | ✓ | | ✓ | | | | | ✓ | ✓ | ✓ |
| Metaxa 2010 [13] | | | ✓ | ✓ | | | | | | |
| Özkan 2004 [23] | | ✓ | | | | | | | | |
| Tremmel 2010 [15] | | | | ✓ | | ✓ | | | | |
| Tutino 2013 [5] | | | | | | | | | | |
| Wu 2015 [6] | | ✓ | | ✓ | | | | | | |
| Wu 2017 [24] | | | | ✓ | | | | | | |
| Yilmaz 2016 [18] | ✓ | | | | | | ✓ | | | |
| Yilmaz 2017 [25] | ✓ | | | | | | | | | |
| **k (studies)** | **7** | **3** | **7** | **5** | **2** | **3** | **2** | **5** | **4** | **3** |
